# Kv3 channel agonist ameliorates the phenotype of a mouse model of amyotrophic lateral sclerosis

**DOI:** 10.1186/s40478-025-02067-z

**Published:** 2025-07-14

**Authors:** Manuela Marabita, Caterina Marchioretti, Aishwarya Aravamudhan, Simona Zito, Antonella Falconieri, Emanuela Zuccaro, Roberta Andreotti, Lisa Gambarotto, Samuele Metti, Marika Tonellato, Valentina Adami, Kyung Ho Park, Martin J. Gunthorpe, Charles H. Large, Agostino Marasco, Sara Vianello, Jessica Rosati, Elisa Belluzzi, Assunta Pozzuoli, Carlo Biz, Pietro Ruggieri, Manuela Basso, Angelo Poletti, Giuseppe Alvaro, Gianni Sorarù, Paolo Bonaldo, Ornella Rossetto, Nadia Pilati, Maria Pennuto

**Affiliations:** 1 BioTiChe Drug Discovery, Istituto di Ricerca Pediatrica Città della Speranza, Padova, 35127 Italy; 2 Department of Biomedical Sciences (DBS), University of Padova, Padova, 35131 Italy; 3 Veneto Institute of Molecular Medicine (VIMM), Padova, 35100 Italy; 4 Department of Molecular Medicine (DMM), University of Padova, Padova, 35131 Italy; 5 Naason Science Inc, Saengmyung-Ro 123, Osong-eup, Heungdeok-gu, Cheongju-si, Chungbuk, 28160 Korea; 6 Autifony Therapeutics, Ltd, Stevenage Bioscience Catalyst, Gunnels Wood Road, Stevenage, SG1 2FX UK; 7 Department of Neuroscience (DNS), Neuromuscular Center, University of Padova, Padova, 35128 Italy; 8 Cellular Reprogramming Unit, Fondazione IRCCS Casa Sollievo della Sofferenza, Foggia, 71100 Italy; 9 UniCamillus - Saint Camillus International University of Health Sciences, Via di Sant’Alessandro, Rome, 8-00131 Italy; 10 Department of Orthopaedics and Orthopaedic Oncology, Department of Surgery, Oncology, and Gastroenterology (DiSCOG), University-Hospital of Padova, Padova, 35128 Italy; 11 Musculoskeletal Pathology and Oncology Laboratory, Department of Surgery, Oncology, and Gastroenterology (DiSCOG), University of Padova, Padova, 35128 Italy; 12 Department of Cellular, Computational and Integrative Biology (CIBIO), University of Trento, Trento, 38123 Italy; 13 Department of Biomolecular and Pharmacological Sciences Rodolfo Paoletti, University of Milan, Milan, 20133 Italy

## Abstract

Voltage-gated potassium channels, Kv3.1, Kv3.2, Kv3.3, and Kv3.4, facilitate rapid repolarization and shape action potentials, which are crucial to maintaining high-frequency firing. Little is known about the expression and function of Kv3 channels in skeletal muscle. We show that these channels are expressed in type IIa/IIx fibers, and their transcript levels progressively increase from postnatal age to adulthood in physiological context. In mature myofibers, the Kv3.1 and Kv3.4 channels are enriched in the muscle triads. The expression of the Kv3 channel is lost upon acute motor unit damage, in mouse models of amyotrophic lateral sclerosis (ALS) and spinal and bulbar muscular atrophy (SBMA), and the skeletal muscle of patients with sporadic ALS. Early treatment of ALS and SBMA mice with AUT00201, a positive allosteric modulator of Kv3 channels, improved the phenotype of ALS mice specifically, suggesting that positive modulation of Kv3 channels is a novel therapeutic option for patients with ALS.

**Supplementary Information:**

The online version contains supplementary material available at 10.1186/s40478-025-02067-z.

## Introduction

Motor neuron and neuromuscular diseases encompass a diverse group of progressive disorders that target upper and lower motor neurons, which are responsible for the contraction of the skeletal muscle, leading to severe impairment in mobility, respiratory function, and overall quality of life. Amyotrophic lateral sclerosis (ALS) is the most prevalent adult-onset motor neuron disease characterized by the degeneration of both upper and lower motor neurons [1, 2]. The genetic complexity of ALS is high, with several causative genes linked to the disorder [3, 4]. Approximately 90% of cases are sporadic, while 10% are familial. The first mutations associated with ALS were found in the superoxide dismutase 1 (*SOD1*) gene, which encodes an enzyme that detoxifies reactive oxygen species and is responsible for around 10% of familial cases [5]. Since then, several genes, including *TARDBP* (TAR DNA Binding Protein), which codes for TDP-43, *FUS*, and *C9ORF72*) have been associated with specific familial forms of ALS, leading to the identification of novel pathogenetic processes involved. Sporadic ALS has a less clearly defined etiology but exhibits similar pathological characteristics, including motor neuron dysfunction and degeneration and skeletal muscle wasting. Spinal and bulbar muscular atrophy (SBMA) is a neuromuscular disease characterized by primary dysfunction and loss of both lower motor neurons and myofibers, leading to progressive muscle weakness, fasciculations and atrophy [6]. SBMA is a monogenic disease caused by expansions of a CAG trinucleotide homorepeat (≥ 38) located at exon 1 of the androgen receptor (*AR*) gene, resulting in the translation of an AR with an aberrantly expanded polyglutamine tract (polyQ) [7]. A key feature of SBMA is its sex discrepancy, with full disease manifestations occurring in males, due to the fact that polyQ-expanded AR is converted to a toxic species upon binding to its natural ligands, namely testosterone and dihydrotestosterone [8]. Despite knowledge of genetic causes, the pathogenetic processes leading to subsequent loss of motor neurons and muscle atrophy in both ALS and SBMA are still poorly understood [9]. These pathogenetic processes are likely to involve a variety of downstream mechanisms affecting the integrity of the motor unit [10–12], leading to hyperexcitability of the motor neuron and innervated myofibers [13–16].

Ion channels play a crucial role in muscle excitability by contributing to maintaining membrane potential, generating electrical signals, and regulating calcium flow through the membrane, thereby linking excitation with muscle contraction. The Kv3 family of voltage-gated potassium channels includes Kv3.1, Kv3.2, Kv3.3, and Kv3.4 and regulates the firing and excitability of neurons and myofibers [17, 18]. Kv3 channels localize to the axons and nerve terminals of neurons within the neocortex, hippocampus, basal ganglia, thalamus, cerebellum and brainstem [19, 20]. Kv3 channels are activated at relatively depolarized membrane potentials in neurons and are distinguished by their very fast activation and deactivation kinetics. These unique biophysical properties enable rapid repolarization of the action potential, which is essential for high-frequency firing. Although the expression and function of Kv3 channels are well characterized in neurons, little is known about other excitable tissues, such as skeletal muscle. In skeletal muscle, Kv3 channels are expressed more in mixed muscles than in fast-twitch muscles [21, 22]. The relevance of muscle expression of these channels emerged with analysis of the phenotype of loss-of-function mouse models and patients carrying pathogenetic mutations in the coding genes. Genetic deletion of the mouse ortholog gene coding for Kv3.1 results in alteration of muscle contraction properties and locomotor activities [23]. Loss of function mutations in a Kv3.4 binding partner in humans are related to periodic paralysis [17], and genetic deletion of the mouse ortholog gene affects muscle contraction and locomotion [24]. However, it is unclear whether alterations in the expression or function of Kv3 channels contribute to acute or chronic damage to the motor unit.

Here, we explored muscle tropism and the age-dependent pattern of expression and function of Kv3 channels in pathophysiological conditions using both mouse genetic models and human muscle biopsies. We obtained evidence that the skeletal muscle expression of the Kv3.1 and Kv3.4 channels is reduced under conditions of acute structural and functional damage to the motor unit, as well as in murine models of ALS and SBMA. These changes in gene expression occurred in the late stage of ALS and in the presymptomatic stage of SBMA. Positive modulation of Kv3 channel activity with a small molecule improved the phenotype of SOD1-G93A mice. On the contrary, similar modulation had no effect on the phenotype of SBMA mice, which is consistent with the early loss of Kv3 channel expression in skeletal muscle. Our findings suggest that stimulation of Kv3 channels represents a novel therapeutic strategy for ALS.

## Results

### The Kv3 channel isoforms are expressed in a fiber type-specific manner with various subcellular localizations at the skeletal muscle junction

**Fig. 1:**
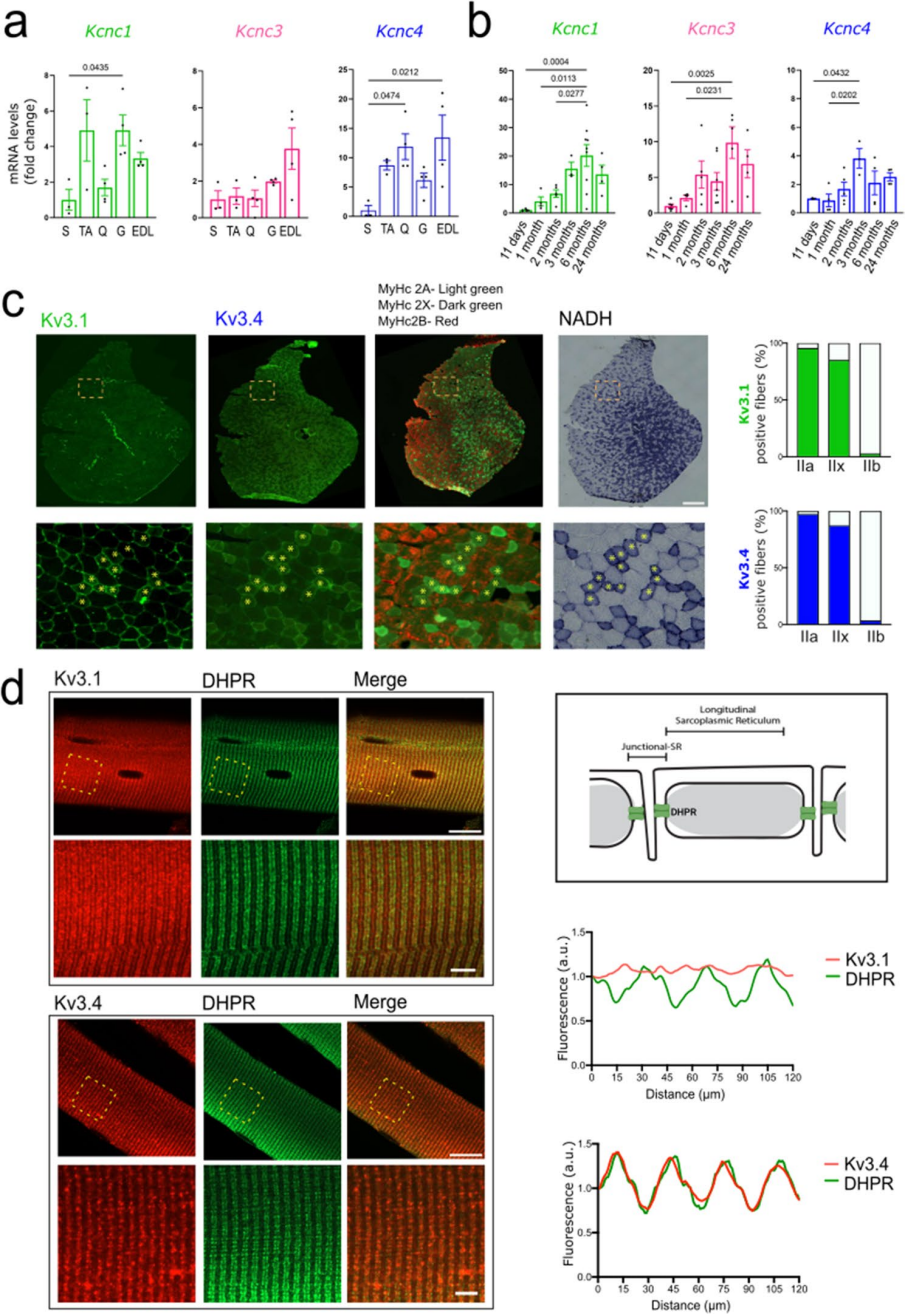
Kv3.1 and Kv3.4 channels are expressed mainly in skeletal muscle type IIa and IIx fibers with a distinct pattern of distribution. **a.** Real-time PCR analysis of the transcript levels of *Kcnc1*, *Kcnc3*, and *Kcnc4* normalized to *beta-actin* in soleus (S), tibialis anterior (TA), quadriceps (Q), and extensor digitorum longus (EDL) muscles derived from 2-month-old wild type male mice (*n* = 3–4 mice) **b.** Real-time PCR analysis of the transcript levels of *Kcnc1*, *Kcnc3*, and *Kcnc4* normalized to *beta-actin* in the TA muscle of wild type male mice at the indicated ages (*n* = 3–9 mice/time point) **c.** Immunofluorescence analysis of Kv3.1 and Kv3.4, MyHC type I (blue), IIa (green), IIx (black), and IIb (red) myofiber subtypes and NADH staining of transversal sections of TA derived from 2-month-old wild type male mice. Upper panels: Entire sections, scale bar: 5 mm. Bottom panels: Magnification of the inset, scale bar: 500 μm. Shown is a representative image (*n* = 3 mice). Right panels: Schematic representation of triads in skeletal muscle fibers. Quantification is shown on the right (type IIa *n* = 342, type IIb *n* = 239, and type IIx *n* = 245 fibers derived from *n* = 3 mice) **d.** Immunofluorescence analysis of fibers isolated from 2-month-old wild type male mice. Upper panels: Fiber sections, scale bar: 25 μm. Bottom panels: Magnification of the inset, scale bar: 10 μm. Quantification is shown on the right (as described in the Methods section). The graphs (**a, b**) show the mean ± SEM; significance was tested with one-way ANOVA followed by Tukey’s HSD test

To characterize the expression pattern of the four members of the Kv3 family in skeletal muscles with different tropism and metabolism, we measured the transcript levels of the *Kcnc1*, *Kcnc2*, *Kcnc3*, and *Kcnc4* genes encoding Kv3.1, Kv3.2, Kv3.3, and Kv3.4, respectively, in different types of muscles derived from wild-type 2-month-old male mice. We analyzed the slow-twitch soleus muscle, which is mainly composed of type I oxidative fibers, and fast-twitch muscles, namely tibialis anterior (TA), quadriceps, gastrocnemius, and extensor digitorum longus (EDL), which are mixed muscles composed of intermediate type IIa and IIx fibers, and in rodents, type IIb glycolytic fibers (Fig. 1a) [25]. By RT-PCR, we did not detect the transcript levels of the *Kcnc2* gene, which is indeed expressed primarily in neurons [19]. Rather, we detected the transcript levels of the *Kcnc1*, *Kcnc3*, and *Kcnc4* genes in all muscle types. In particular, the transcript levels of *Kcnc1* and *Kcnc4* were higher in the EDL, TA, gastrocnemius, and quadriceps muscles compared to soleus. Subsequently, we investigated whether the expression of these channels varies with age (Fig. 1b). We found a gradual increase in the expression of the *Kcnc1*, *Kcnc3*, and *Kcnc4* genes from postnatal day 11 through adulthood. Expression remained high up to 2 years of age for the three channels, suggesting stable expression during adulthood in wild-type mice.

We then asked whether Kv3 channels are expressed in different fiber subtypes (Fig. 1c). We processed contiguous transversal sections of TA muscles from 2-month-old wild-type mice for immunofluorescence analysis of specific fiber subtype, that is, myosin heavy chain (MyHC) IIa, MyHCIIx, and MyHC IIb, coupled with NADH staining to merge information with fiber metabolism (oxidative versus glycolytic). Although we did not find an antibody for Kv3.3 suitable for this analysis, we set out a protocol for specific staining for Kv3.1 and Kv3.4. We found that Kv3.1 and Kv3.4 are expressed mainly in type IIa and IIx fibers compared to type IIb fibers. Next, we sought to analyze the localization of Kv3.1 and Kv3.4 in single longitudinal myofibers of TA muscles (Fig. 1d), as previously described [26]. We combined Kv3.1 or Kv3.4 staining with dihydropyridine receptors (DHPRs) that are enriched in the junctional sarcoplasmic reticulum (J-SR), which is the region of the terminal SR cisternae that faces the t-tubules in triads between longitudinal SR (L-SR). Using confocal microscopy and assessing the fluorescence intensity profile, we found that Kv3.4 has a regular doublet pattern that overlaps that of DHPR, indicating that Kv3.4 is mainly localized at the muscle triads, while Kv3.1 is enriched, but not exclusive to the triads.

### Expression of Kv3 channels is highly responsive to acute motor unit damage

**Fig. 2:**
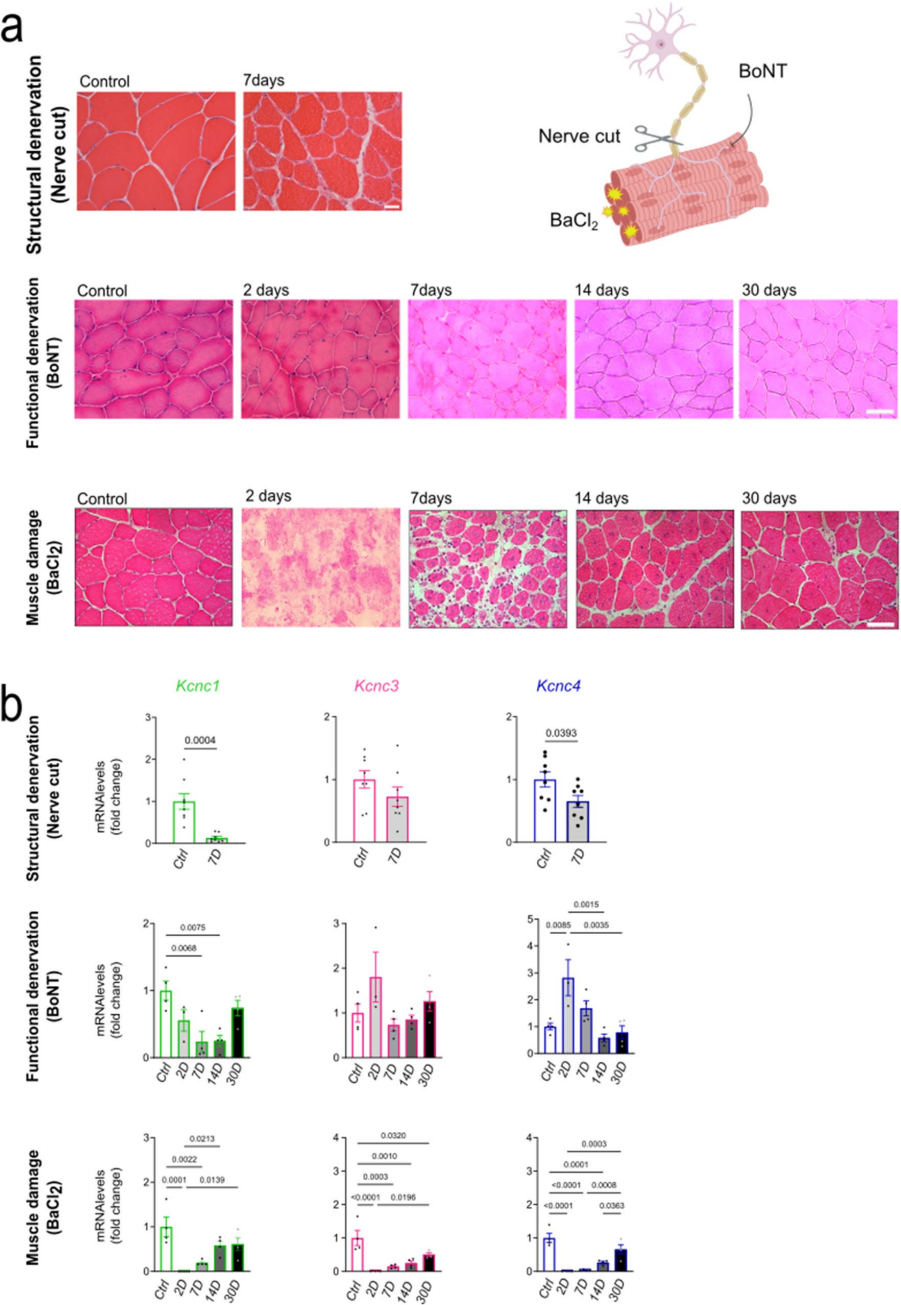
Kv3 channel expression is decreased upon acute damage of the motor unit. **a.** Right panel: Schematic representation of treatment using nerve cut, BoNT and BaCl_2_. Hematoxylin-eosin staining of transversal sections of TA muscles of 3-month-old wild type male mice. Shown is a representative image (*n* = 3 mice). Bar, 50 μm **b.** Real-time PCR analysis of the transcript levels of *Kcnc1*, *Kcnc3*, and *Kcnc4* genes normalized to *beta-actin* in the TA at the indicated time points (*n* = 3–7 mice). The graphs show the mean ± SEM; significance was tested with one-way ANOVA followed by Tukey’s HSD test or by the two-tailed Student’s t-test

Since muscle expression of the Kv3.4 channel is sensitive to denervation [22], we asked whether acute damage of the motor unit affects the expression of all family members. To dissect the impact of motor neuron and myofiber damage and their communication, we employed three experimental paradigms: (i) Sciatic nerve resection to cause structural denervation, (ii) Botulinum neurotoxin (BoNT) injection to cause a reversible functional denervation without structural disassembly of the NMJ [27], and (iii) Injection of barium chloride (BaCl_2_) to directly induce extensive myofiber degeneration [28] (Fig. 2a). We analyzed muscle pathology at various time points. By hematoxylin/eosin staining of transversal sections of TA muscle, we found that nerve cut and BoNT injection cause atrophy, whereas BaCl_2_ injection causes massive damage followed by progressive formation of centronucleated fibers, which is a sign of muscle regeneration (Fig. 2a). Then, we analyzed the expression levels of genes associated with muscle denervation such as the acetylcholine receptor (*Chrn*), myogenin (*Myog*), and Runt-related transcription factor 1 (*Runx1*), and muscle regeneration such as *Myhc3* and *Myhc8* (Suppl. Figure 1a). As expected, nerve resection and BoNT injection resulted in upregulation of denervation and muscle regeneration markers, whereas BaCl_2_ injection resulted in upregulation of mainly muscle regeneration markers. Then we assessed the levels of expression of the genes that encode the Kv3 channels (Fig. 2b, Suppl. Figure 1b). The *Kcnc1* transcripts were down-regulated after structural and functional denervation as well as muscle damage, indicating that the expression of Kv3.1 channels is sensitive to the integrity of the motor unit. The levels of the *Kcnc3* transcript were negatively regulated with muscle damage, while the *Kcnc4* transcripts decreased after nerve resection and BaCl_2_ injection, but not after injection of BoNT, indicating that the expression of this channel is modified with structural denervation and muscle damage. Interestingly, the expression of these genes gradually returned back to normal along with the myofiber regeneration process. These results indicate that the transcription of the genes encoding Kv3.1, Kv3.3, and Kv3.4 is sensitive to acute damage to the motor unit.

### Down-regulation of Kv3 channels in chronic mouse models of motor neuron and neuromuscular diseases

**Fig. 3:**
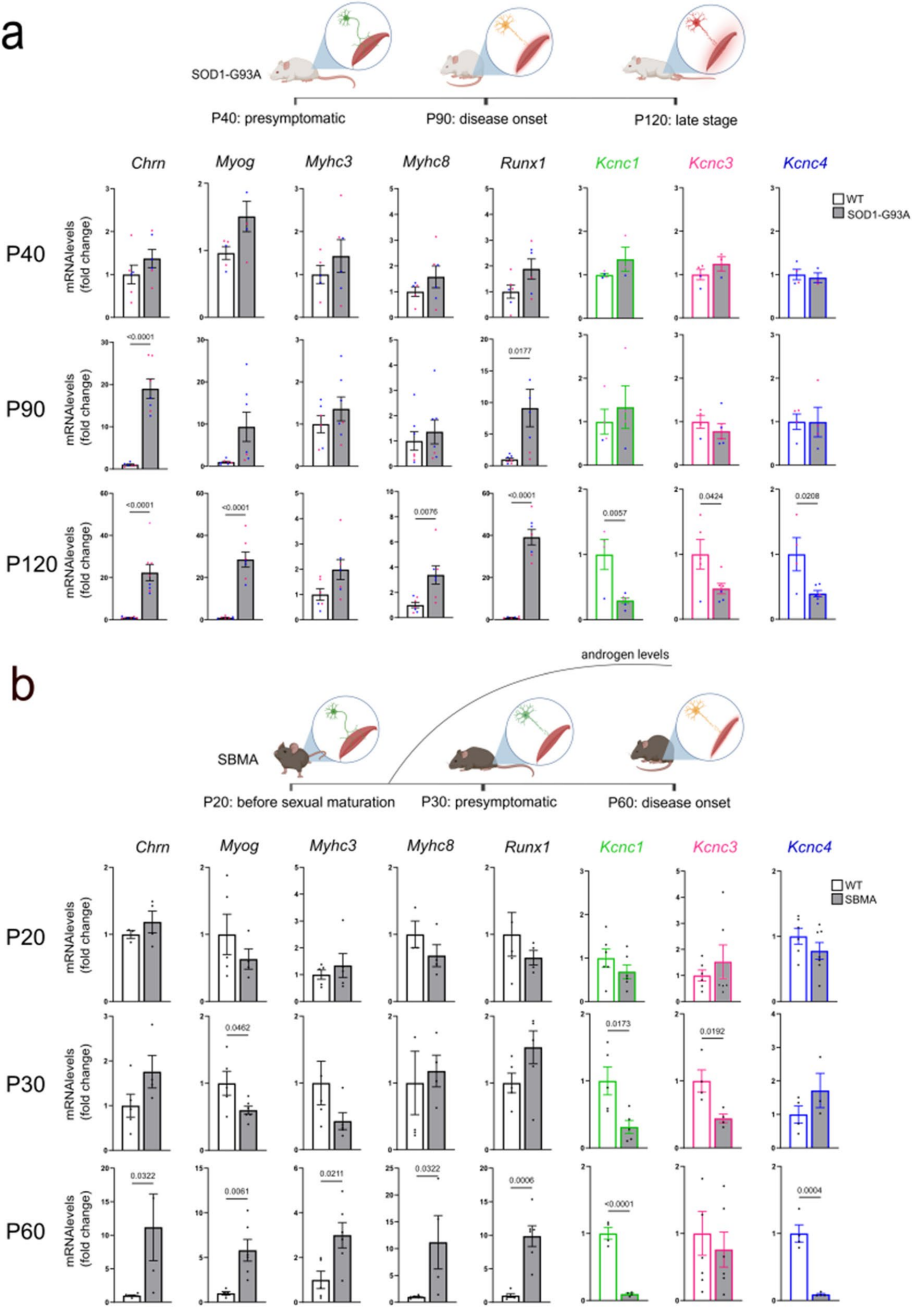
Kv3 channel expression is decreased in chronic models of motor neuron and neuromuscular diseases. **a-b**) Upper panels: Scheme of disease stage in the SOD1-G93A mice modelling ALS (**A**) and AR100Q mice modeling SBMA (**B**). Bottom panels: Real-time PCR analysis of the transcript levels of the indicated denervation and muscle atrophy genes (grey) and *Kcnc1*, *Kcnc3*, and *Kcnc4* genes (color code) in transgenic male (blue dots) and female (pink dots) mice expressing SOD1-G93A modelling ALS (**A**, *n* = 3–7 mice), and transgenic male mice expressing AR100Q modelling SBMA (**B**, *n* = 3–7 mice). The graphs show the mean ± SEM; significance was tested with the two-tailed Student’s t-test

Given the above observations, we asked whether the expression of Kv3 channels is affected by adult-onset chronic degenerative disorders, namely ALS and SBMA. To model ALS, we used transgenic mice expressing SOD1 with glycine 93 mutated to alanine (SOD1-G93A) (Fig. 3a, **top panel**) [29], and to model SBMA we used transgenic mice expressing AR with a polyQ tract of 100 glutamine residues (AR100Q) (Fig. 3b, **top panel**) [26, 30]. We analyzed three stages of the disease, corresponding to 40 days of age (presymptomatic), 90 days of age (onset of the disease, defined as the onset of motor dysfunction) and 120 days of age (late stage) in SOD1-G93A mice, and 30 days of age (presymptomatic stage) and 60 days of age (onset of the disease, also here defined as the onset of motor dysfunction) in AR100Q mice. For SBMA that is strictly an androgen-dependent disease [8], we also analyzed an earlier time point, i.e., 20 days of age, before sexual maturation [31]. Both muscle denervation and regeneration markers were up-regulated in the TA muscle of ALS (Fig. 3a, **left panels**) and SBMA (Fig. 3b, **left panels**) mice at the age of the onset of motor dysfunction. In ALS mice, the transcript levels of the *Kcnc1*, *Kcnc3* and *Kcnc4* genes were down-regulated in a late stage (Fig. 3a, **right panels**). In SBMA mice, the transcript levels of *Kcnc1* and *Kcnc3* were down-regulated in the presymptomatic stage, and *Kcnc4* at the onset of motor dysfunction (Fig. 3b, **right panels**). These latter gene expression changes in AR100Q mice were concomitant with the natural increase in androgen levels that occurs around 30 days of age or afterward. These observations show that the expression of genes coding for Kv3 channels is altered in the skeletal muscle of neurodegenerative diseases, at a late stage in ALS and at a presymptomatic and symptomatic stage in SBMA.

### AUT00201 is a positive modulator of Kv3 channels

**Fig. 4:**
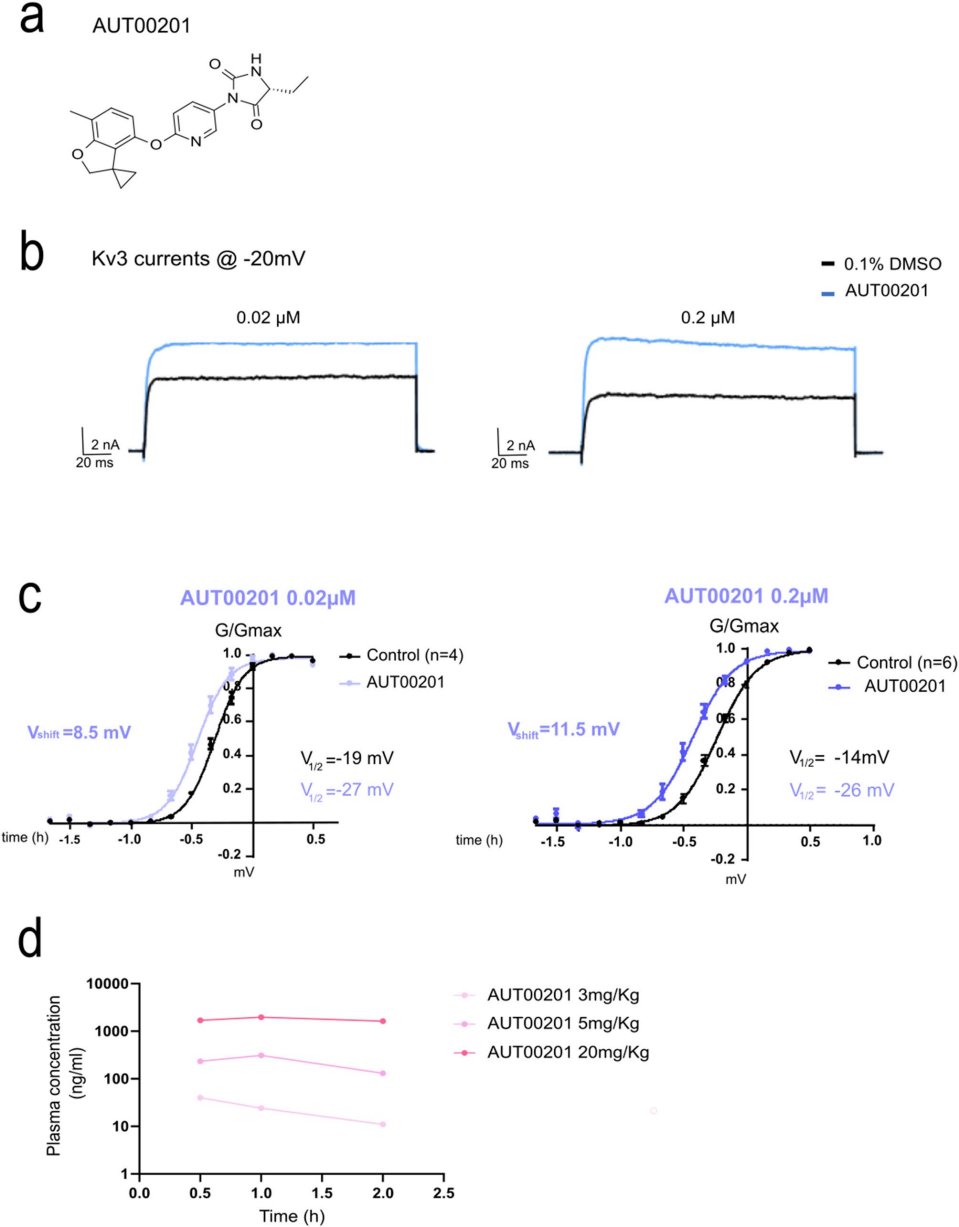
AUT00201 positively modulates Kv3 channel currents. **a.** Chemical structure of AUT00201 **b.** Representative traces of human Kv3.1 mediated currents in HEK293Tcells. Currents were evoked by voltage steps to -25mV before (black) and after (blue) treatment with AUT00201 (0.02 and 0.2µM) **c.** The effects of AUT00201 on the activation voltage-dependence were evaluated by plotting normalized conductance as a function of membrane voltages. AUT00201 produced a concentration-dependent shift on the activation of Kv3.1 channels to more hyperpolarized potentials **d.** Plasma concentration of AUT00201 upon treatment of wild type male mice (*n* = 3 mice/group)

AUT00201 ((5R)-5-ethyl-3-[6-(7-methylspiro[2 H-benzofuran-3,1’-cyclopropane]-4-yl)oxy-3-pyridyl]imidazolidine-2,4-dione) is a novel small molecule that selectively improves the activity of Kv3 channels (Fig. 4a), derived from previously validated channel modulators [32, 33]. To validate the positive modulator properties of AUT00201, we expressed human Kv3.1 channels in human embryo kidney (HEK) 293T cells and treated the cells with two concentrations of compound, that is, 0.02 µM and 0.2 µM (Fig. 4b). Using a whole cell patch clamp, we measured Kv3.1 currents evoked by depolarising the membrane from a holding potential of -90 mV to test potentials from − 100 mV to + 30 mV in increments of 10 mV. Treatment significantly increased Kv3.1 currents, especially at voltages close to the threshold for Kv3 channel activation (-40 to 0 mV). When the normalised conductance was plotted as a function of membrane voltage, there was a shift to the left in the voltage dependence of activation upon compound treatment (Fig. 4c). Thus, AUT00201 modulates the voltage dependence of activation of Kv3.1 channels and lowers the voltage at which the channels are activated, thus facilitating channel opening at more hyperpolarized potentials. Similarly, using an Ionworks TM automated patch-clamp assay, AUT00201 increased the Kv3.1, Kv3.2 and Kv3.4 currents measured at -15mV in a concentration-dependent manner with pEC50 values of 6.15 ± 0.04, 6.52 ± 0.05, and 5.6 ± 0.04, respectively, indicating that AUT00201 also modulates human Kv3.2 and Kv3.4 channels with different potencies. Next, we assessed the pharmacokinetics of AUT00201 administered orally to wild-type mice. AUT00201 is characterized by favorable physicochemical properties with good overall cell and brain permeability (brain/plasma = 1). We observed a rapid increase in plasma concentrations after Cmax dosing achieved 30–60 min after administration and relatively stable levels maintained for up to 120 min. In detail, AUT00201 reached a plasma maximum concentration of 40, 310, and 1970 ng/ml after oral doses of 3, 5, and 20 mg/kg (Fig. 4d), respectively, corresponding to a brain/plasma free concentration of 0.002, 0.017 and 0.1 µM, given 98% protein binding. Since in the in vitro electrophysiology experiments we observed effects of AUT00201 on different subtypes of the Kv3 channel at concentrations of 0.02 µM and above, we selected doses of 5 mg / kg (low dose) and 20 mg / kg (high dose), corresponding to a free concentration of 0.017 and 0.1 µM for further in vivo studies.

### Treatment of SOD1-G93A mice with AUT00201 attenuates motor dysfunction, motor neuron loss and muscle pathology

**Fig. 5:**
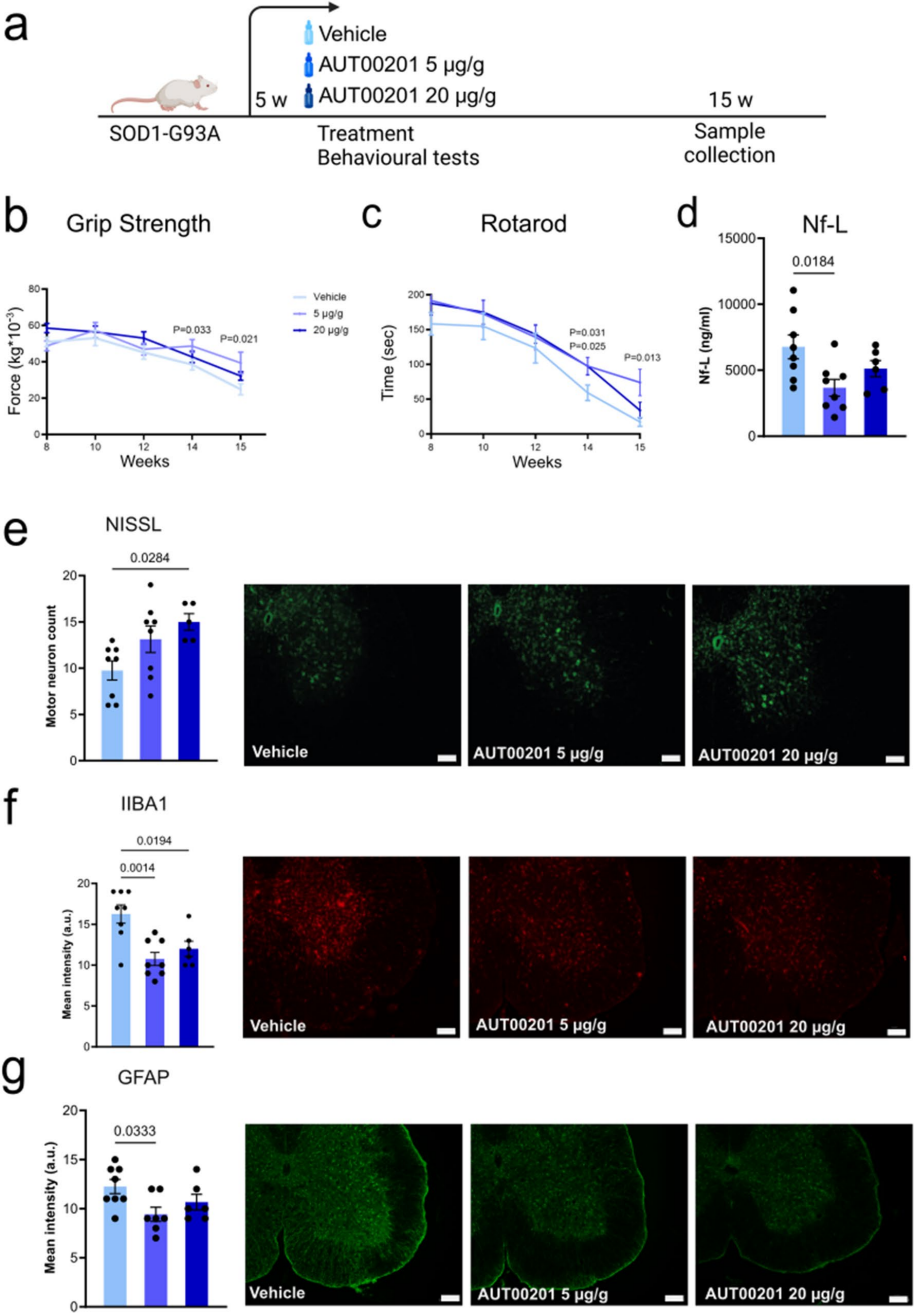
Treatment with an agonist of Kv3 channels attenuates motor dysfunction, motor neuron loss and muscle pathology in SOD1-G93A mice **a.** Scheme of treatment and behavioral tests **b.** Grip strength analysis of muscle force of SOD1-G93A mice treated with either vehicle or AUT00201 (*n* = 8 mice/group) **c.** Rotarod task analysis of motor coordination of SOD1-G93A mice treated with either vehicle or AUT00201 (*n* = 8 mice/group) **d.** Nf-L serum concentration of SOD1-G93A mice treated with either vehicle or AUT00201 (*n* = 8 mice/group) **e.** Nissl staining analysis of motor neuron number in the lumbar spinal cord transversal sections of SOD1-G93A mice at 15 weeks of age (*n* = 8 mice/group). Scalebar, 100 μm **f.** IBA1 staining in the lumbar spinal cord transversal sections of SOD1-G93A mice at 15 weeks of age (*n* = 8 mice/group). Scalebar, 100 μm **g.** GFAP staining in the lumbar spinal cord transversal sections of SOD1-G93A mice at 15 weeks of age (*n* = 8 mice/group). Scalebar, 100 μm. The graphs show the mean ± SEM; significance was tested using two-way (**b, c**) and (**d-g**) one-way ANOVA followed by LSD post-hoc analysis or by the one-tailed Student’s t-test. Shown are representative images

Next, we tested whether positive modulation of Kv3 channel activity with AUT00201 delays or prevents the onset and progression of symptoms of ALS and SBMA mice. Given the observation that the expression of the Kv3 channel is preserved during the early stages of disease manifestations, we started treatment at time points at which Kv3 channel expression is normal, that is, 5 weeks of age for SOD1-G93A mice (Fig. 5a) and 20 days of age for AR100Q mice (Suppl. Figure 2a) with AUT00201 (5 and 20 mg / kg). To assess the effect of treatment on motor neuron function and muscle strength, we measured muscle force by grip strength task and motor coordination by rotarod task. AUT00201 did not modify the phenotype of AR100Q mice (Suppl. Figure 2b-c). Although treatment of SOD1-G93A mice with 5 and 20 mg / kg AUT00201 did not modify lifespan (Suppl. Figure 3a), it produced a modest, but significant increase in muscle force and motor coordination at 8 and 12 weeks of age for the high dose and 14 weeks of age for the low dose (Fig. 5b-c). Neurofilament light chain (NF-L) is a clinical biomarker of ALS that correlates with disease progression [34]. Treatment of SOD1-G93A mice with low-dose AUT00201 resulted in a significant decrease by 45% of serum NF-L levels (Fig. 5d). Although treatment did not modify neuromuscular junction pathology (Suppl. Figure 3b-d), treatment was associated with a significant increase in the number of motor neurons by 1.3-fold in the ventral spinal cord compared to vehicle-treated mice (Fig. 5e). A key component of ALS pathology is the extensive and chronic activation of microglia [35], and gliosis [36, 37]. As microglia and astrocytes actively contribute to different aspects of ALS, namely disease onset and progression, respectively [38, 39], we sought to determine whether treatment of mice with AUT00201 attenuates the activation of these cell types. Thus, we performed immunofluorescence analysis of ionized calcium-binding adapter molecule 1 (IBA1), which is a marker of activated microglia, and glial fibrillary acidic protein (GFAP), which is a marker of activated astrocytes, in transversal sections of the gastrocnemius muscle of 15-week-old SOD1-G93A mice treated with vehicle and AUT00201 (Fig. 5f-g). Treatment significantly decreased the fluorescence intensity of IBA1 and GFAP staining by 10–20% in mice treated with AUT00201 compared to mice treated with vehicle, indicating that AUT00201 reduces microglia activation and gliosis in this murine model of ALS.

### Deregulation of Kv3.4 expression in the skeletal muscle of sporadic patients with ALS

**Fig. 6:**
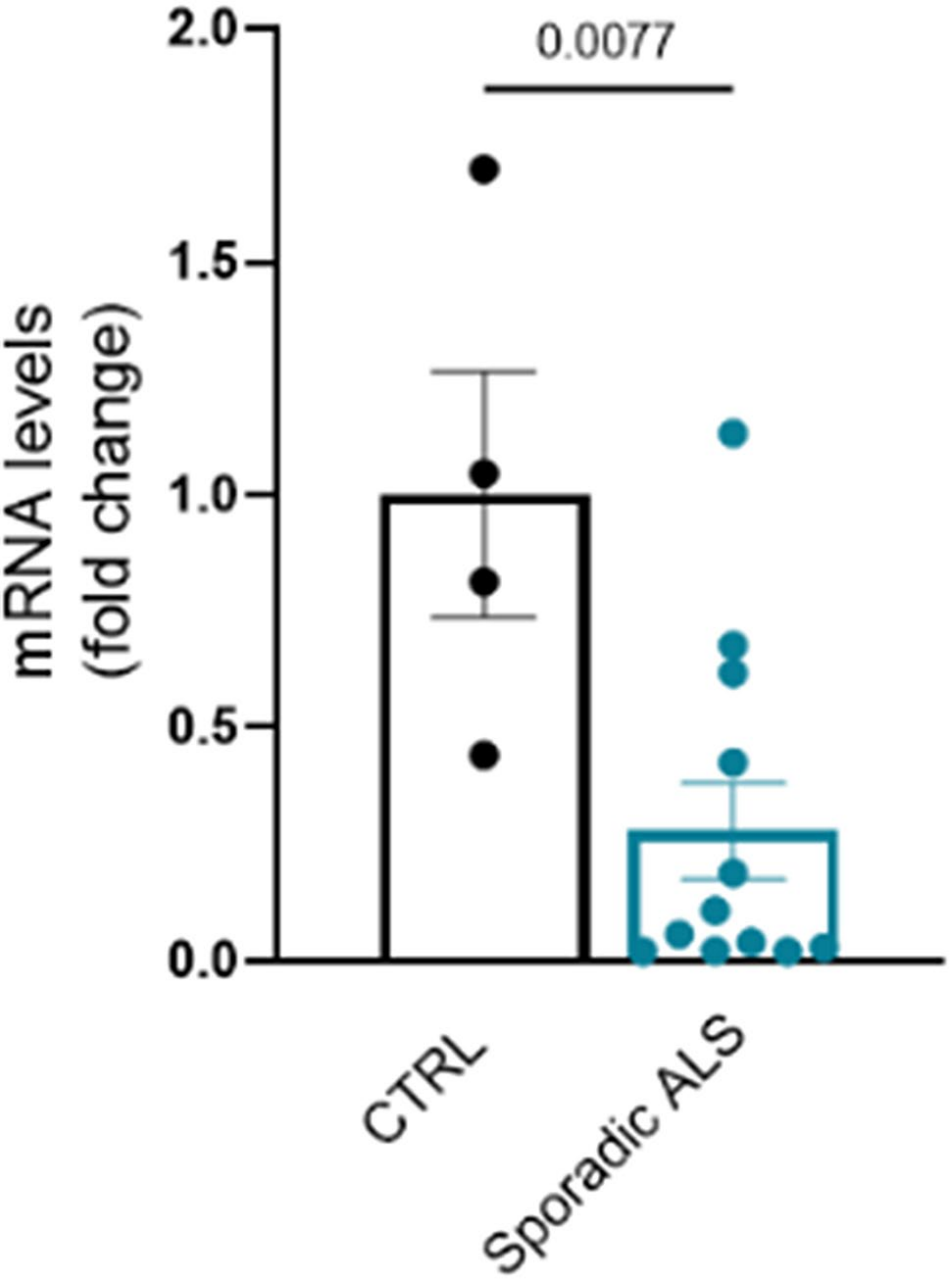
*Kcnc4* gene is downregulated in the skeletal muscle of patients suffering from sporadic ALS. Real-time PCR analysis of the transcript levels of *Kcnc4* normalized to *beta-actin* in human muscle biopsies from sporadic ALS patients and healthy controls (*n* = 4–12). The graphs show the mean ± SEM; significance was tested by the two-tailed Student’s t-test

To assess the disease relevance of our findings, we tested whether the expression of the Kv3 family channels is altered in skeletal muscle of ALS patients. Although we used several pairs of primers, we could not detect the transcript levels of the *KCNC1* gene in the *vastus lateralis* muscle samples. By RT-PCR, we measured the transcript levels of the *KCNC4* gene. We found that the expression of the Kv3.4 channel was significantly reduced by 72% in the skeletal muscle of sporadic ALS patients compared to healthy subjects of the same age (Fig. 6). Additionally, we analyzed the transcript levels of the *KCNC4* gene in ALS patients carrying mutations in *FUS*, *C9orf72*, and *TARDBP (*TDP-43*)*; however, the small sample size did not allow for significant results (Suppl. Figure 4).

In conclusion, our data indicate that changes in Kv3 channel expression occur in skeletal muscle after acute and chronic damage to the motor unit, as well as in the muscle of patients with sporadic ALS, and treatment targeting these channels before their loss may be a valuable therapeutic strategy for patients.

## Discussion

Here, we show that Kv3 channels are important constituents of striated muscles. Attempts to impact the progression of neuromuscular degeneration in SBMA and ALS mice with the positive modulator of the Kv3 channel, AUT00201, revealed some benefits in the case of the ALS model. This finding, together with the observation that Kv3.4 channel expression is down-regulated in the muscle of ALS sporadic patients, paves the way for the development of early treatment targeting this family of potassium channels to alleviate symptoms.

The present study was designed to explore the expression of Kv3 channels in striated muscle in healthy tissue and in acute and chronic conditions that affect the motor unit. First, we showed that Kv3.1, Kv3.3 and Kv3.4 are expressed in mouse striated muscles with a pattern of expression increasing from postnatal age through adulthood, whereas Kv3.2 channels do not appear to be expressed by muscle, at least in the mouse. Our evaluation of expression in human muscle samples confirmed the expression of Kv3.4 channels. In terms of localization within specific muscle types, the channels are predominantly expressed in type IIa and IIx fibers. The presence of Kv3 channels only on the fast fibers is reminiscent of the selective expression of the channels on “fast firing” neurons [20]. This suggests a physiological role for Kv3 channels in supporting rapid muscle contraction. At the subcellular levels, the Kv3.1 channels are uniformly distributed along the sarcolemma, while the Kv3.4 channels are enriched in the muscle triads. Another aspect that should be taken into account is that the key players required for controlling the Kv3 channel function are their binding partners, which modulate the channel function. One such binding partner is MinK-related peptide 2 (MiRP2), which controls Kv3.1 function in neurons [40]. MiRP2 plays a key role also in muscle contraction, as underlined by the fact that loss-of-function mutations of MiRP2 are associated with familial periodic paralysis [17]. Furthermore, genetic deletion of the gene coding for MiRP2 in mouse results in increased oxidative metabolism, abnormal hind-limb clasping and altered muscle contraction, further providing evidence that the complete asset of Kv3 channel subunits and binding partners are required for normal skeletal muscle function [24]. Future investigations will clarify the role and impact of MiRP2 on the function of the KV3 channel in skeletal muscles subject to acute and chronic damage.

Dysregulation of Kv3 channel expression or function has been implicated in several neurological diseases, spanning from periodic paralysis [17] to spinocerebellar ataxia [41] and epilepsy [42]. Moreover, Kv3.4 expression is reduced in Huntington’s disease [43], which similar to SBMA is caused by exonic CAG expansions in the gene coding for huntingtin [44]. Kv3 potassium channels may indeed have a broad relevance across the family of neurodegenerative diseases, which also includes Alzheimer’s disease and Parkinson’s disease [45]. Any damage to the motor neuron, the innervated myofibers and the surrounding cell types disrupts the homeostasis and function of the motor unit through both cell-autonomous and non-cell autonomous mechanisms [38, 39, 46]. The composition and characteristics of skeletal muscle fibers are influenced by changes in gene expression that occur during development and maturation under normal physiological conditions, as well as in response to acute and chronic damage. Notably, genes such as parvalbumin and other genes controlling muscle contraction [26], chloride channel type 1 [47], and voltage-gated Kv channels, as shown here, undergo significant alterations in a pathological setting. A key finding here was that the expression of Kv3 channels is sensitive to acute damage of the motor unit and chronic motor neuron and neuromuscular diseases, such as ALS and SBMA. Interestingly, dysregulation of Kv3 channel expression was an early event in SBMA mice and a late event in ALS mice, likely resulting from different pathological processes occurring in these two diseases of the motor unit. Kv3.1 expression may be a sensitive marker of disease progression in the AR100Q mouse and perhaps can potentially play a role in the pathophysiological process. However, *Kcnc1* and *Kcnc4* early down-regulation in the SBMA muscle imposes a limit to treatment/efficacy. The lack of efficacy is likely linked to the early loss of Kv3 channels in SBMA muscle, leaving no channels available to modulate. As a result, treatment with Kv3 channel agonists alone was not sufficient to ameliorate the progression of disease manifestations. This does not exclude the possibility that administration of the Kv3 channel agonist together with additional medications, such as leuprorelin [48], may have beneficial effects. Based on current knowledge on the role of Kv3 channels in skeletal muscle, it is important to underline that the early Kv3 gene expression changes observed in SBMA muscle are unlikely to be harmless. Moreover, our results suggest that these channels are regulated by specific signaling pathways lost or altered upon acute degeneration of the motor unit. Further research will clarify the signaling pathways through which the motor neuron and the innervated myofibers communicate with each other, and how this is linked to Kv3 channel expression.

Different from SBMA, all the three Kv3 subtypes showed a significant reduction only at the advanced stages of degeneration in the SOD1-G93A mouse model (P120). This gave us the possibility to administer the compound long before the downregulation of Kv3 channel expression, and as a matter of fact, treatment with AUT00201 produced modest but significant effects on the SOD1-G93A phenotype that were consistent across behavioral and histological endpoints. Treatment ameliorated muscle force, indicating that AUT00201 attenuates muscle weakness. This effect was associated with different beneficial effects on neuromuscular pathology. By Nissl staining analysis, AUT00201 significantly reduced motor neuron loss in the ventral spinal cord of ALS mice. A key finding was the effect of treatment on Nf-L. Neurofilaments, key components of the neuronal cytoskeleton, play a crucial role in maintaining axonal structure and transport. Aberrant neurofilament dynamics represent hallmark pathological signs in ALS [49, 50]. Elevated levels of phosphorylated neurofilament heavy chain (pNf-H) and Nf-L are consistently detected in the cerebrospinal fluid and blood of ALS patients, reflecting axonal damage and neuronal degeneration [51, 52]. These biomarkers not only aid in diagnosing ALS but also correlate with disease progression and severity, offering prognostic value [53–55]. Pathologically, neurofilament accumulation and mislocalization within motor neurons contribute to axonal transport deficits and cellular stress, exacerbating neurodegeneration. Understanding the link between correct expression of Kv3 channels and neurofilament dysregulation in ALS may provide insights into disease mechanisms and underscore their potential as therapeutic targets and biomarkers for monitoring disease progression. Another important effect of treatment was on microglia activation and gliosis, which are prominent features of and play a significant role in disease progression. Microglia, the resident immune cells of the central nervous system, become activated in response to motor neuron degeneration, transitioning to a pro-inflammatory phenotype that exacerbates neuronal damage through the release of cytokines [56, 57]. This activation is accompanied by astrogliosis, where astrocytes proliferate and release neurotoxic factors, further amplifying neuroinflammation and motor neuron loss [58, 59]. The effect of treatment on microglia and astrocyte activation implies a beneficial effect on these aspects of pathology linked to ALS. Our data show that the low dose is the most effective one. This is not unusual for ion channel modulators, where only a small proportion of the current needs to be modulated to achieve a therapeutic effect. Higher levels of modulation can lead to overactivation of the channel, potentially resulting in reduced efficacy or off-target effects. AUT00201 has already undergone Phase I clinical trial (NCT05873062). Thus, this approach may be soon tested in additional mouse models of ALS and ultimately in patients.

Finally, we show here that Kv3.4 expression is reduced in striated muscle tissue obtained from patients suffering from ALS, compared to healthy control subjects. These results were obtained mostly in sporadic patients, and as such they are important for two reasons. First, they show Kv3 channel gene expression changes in patients suffering from the most frequent form of ALS. Second, they suggest novel molecular biomarkers that can help establish the efficacy of treatment. Further research is needed to confirm the expression and role of other Kv3 channel isoforms in human skeletal muscle. Kv3 positive modulators, such as AUT00201, show promise as potential ALS treatments, either alone or combined with other therapies, and may be effective at various stages of the disease.

## Methods

### Animals

Male SOD1*G93A (High-copy SOD1*G93A, G1H with 25 TG copies; stock# 002726) and female B6SJL strain background (non-carrier; wild type) obtained from Jackson Laboratories through Raon Bio (Korea) were mated to generate F1 mice for the experiment. SBMA transgenic mice expressing AR with 100 glutamine residues (AR100Q) were maintained in a congenic C57Bl6J background, as previously described [26, 30]. All mice were housed in a temperature (22 ± 1 °C) and humidity (30–50%) controlled environment with a normal light-dark cycle (8:00–20:00). Food and water were available *ad libitum* to the mice in their home cages. Mice were monitored daily by specialized operators and by the designated veterinary.

### Compound delivery and formulation

AUT00201 was provided by Autifony and prepared according to the Autifony’s guideline and recommendation. The operator was double-blind for genotype and treatment. To mitigate potential bias, participants and the researchers involved in the data analysis were also double-blind. For the treatment of SOD1-G93A mice, the vehicle solution consisted of Gelucire 44/14, PEG400, and water in a 5:3:2 ratio. AUT00201 was administered to male SOD1 transgenic and WT mice once a day via oral gavage (P.O.) starting at 5 weeks of age. Meanwhile, in SBMA mice, AUT00201 was formulated in a condensed milk/PEG400 mixture (4:1) and administered orally via dropper daily.

### Behavioral tests

#### Rotarod

Mice were tested during the diurnal phase of the day. Testing was performed every week until the end of the study. On the test day, a training trial of 5 min at 4 rpm on the rotarod apparatus was performed. One hour later, the animals were tested for two consecutive accelerating trials of 6 min with the speed changing from 0 to 40 rpm over 360 s and an inter-trial interval of at least 30 min. The latency to fall from the rod was recorded. Mice remaining on the rod for more than 360 s are removed and their time scored as 360 s.

#### Grip strength test

Grip strength measurement was performed every week until end of the study. Mice were placed on the grip-strength apparatus (San Diego Instruments, San Diego, USA) in such a way that the animal grabbed a small mesh grip with its forepaws. Animals were lowered to the platform and then slowly pulled away from the handle by the tail until the animal released the handle. The equipment automatically measured the strength of the animal’s grip in grams. One day session included 5 scores per animal that was recorded in a consecutive sequence.

### Human samples

Anonymized control biopsies of healthy muscle were collected as residual biological samples during surgery from patients with femur fractures. Patient biopsy samples were obtained from the Neuromuscular Bank of Tissues and DNA Samples, Telethon Network of Genetic Biobanks, and of the EuroBioBank Network. Muscle biopsies were collected for diagnostic purposes, with written informed consent obtained from each patient in compliance with the Helsinki Declaration. All patients who underwent muscle biopsy were clinically affected and showed weakness and/or muscle atrophy. Detailed information, including age, biopsied muscle, clinical status of controls, and Q tract length in SBMA patients, is provided in Supplementary Table 1.

### Quantitative real-time PCR analysis

Total RNA was extracted with TRIzol (Thermo Fisher Scientific), and RNA was reverse transcribed using iScript Reverse Transcription Supermix (1708841 Bio-Rad) following the manufacturer’s instructions. Gene expression was measured by RT-qPCR using the SsoAdvanced Universal Sybr green supermix (1725274 Bio-Rad) and the C1000 Touch Thermal Cycler-CFX96 Real-Time System (Bio-Rad). Gene expression was normalized to actin expression levels. The complete list of materials and primer sequences are provided in Supplementary Table 2.

### Immunofluorescence and histological staining

#### Nissl staining

Ventral horn motor neurons from the lumbar samples (20-µm thick sections cut between L1-L3 of lumbar levels of spinal cord) were counted via immunofluorescence using NeuroTrace (Nissl stain, Thermo fisher). Lumbar-1 samples were sectioned with a thickness of 20 μm. Three slices per lumbar-1 were mounted on a microscope slide. Each slide was washed in PBS solution and permeabilized with 0.1% Triton X-100 in PBS for 10 min at room temperature. The NeuroTrace was subjected to 1:300 dilution and allowed to stand at room temperature for 20 min. After washing the sample with PBS, it was completely dried and mounted with a mounting solution containing DAPI.

The motor neuron-stained images were visualized using the Olympus’s Immunofluorescence microscope (Olympus, BZ21). The number of motor neurons in the ventral horn of the lumbar was counted. Only the cells of sizes of 750 pixels or larger were counted using the Image J software.

#### GFAP/Iba1 staining

Immunofluorescence analysis of the ventral horn motor neurons was performed using GFAP (Cell signaling technology) for astrogliosis, and Iba1 (WAKO) for microgliosis. Lumbar samples were sectioned with a thickness of 20 μm. Three slices per lumbar were mounted on a microscope slide. The slide was washed in PBS solution and blocked in a solution containing PBS, 5% normal goat serum and 0.3% Triton X-100 for 1 h at RT. The sections were incubated overnight at 4 °C with the appropriate primary antibody. The sections were washed in PBS solution, and incubated in secondary antibody for 1 h at RT. Finally. the sections were washed in PBS solution and completely dried and mounted with a mounting solution containing DAPI. The astrogliosis and microgliosis stained images were visualized using the Olympus’s Immunofluorescence microscope (Olympus, BZ21). The intensity of fluorescence in the ventral horn of the lumbar was analyzed using the Image J software.

#### Neuromuscular junction staining

For evaluation of neuromuscular junctions (NMJ) and muscular innervation, immunohistochemical analyses using anti-synaptotagmin-2 (pre-synaptic marker, SV2, DSHB) and a-bungarotoxin (post-synaptic marker) were performed on gastrocnemius muscle samples. Gastrocnemius muscle samples were sectioned with a thickness of 20 μm. One slice per gastrocnemius muscle was mounted on a microscope slide. The slides were washed in TBS solution. The sections were then permeabilized for 15 min in TBST, rinsed twice in TBS and blocked in a solution containing TBS, 5% normal goat serum and 0.2% Triton X-100 for 1 h at RT. The sections were incubated overnight at 4 °C with the appropriate primary antibodies. The sections were washed in TBS solution, and incubated in secondary antibody for 1 h at RT. Finally, the sections were washed in TBS solution and completely dried and mounted with a mounting solution containing DAPI. The NMJ stained images were visualized using a confocal microscope (Lecia, Thunder). For analysis, the degree of overlap between the SV2 (pre-synaptic) and α-bungarotoxin (post-synaptic) regions was classified into three different categories: ‘Fully-innervated’ for completely overlapping, ‘partially innervated’ for partial overlapping and ‘denervated’ for non-overlapping pre-synaptic and post-synaptic regions.

#### Neurofilament light chain (Nf-L) measurement in plasma

Nf-L concentration in the mouse plasma was determined using the Simoa Nf-L assay. Paramagnetic carboxylated beads (Quanterix Corp, Boston, MA, USA) were coated with a mouse anti-neurofilament light antibody and incubated for 35 min with a sample and a biotinylated mouse anti–neurofilament light antibody in the Simoa instrument (Quanterix). The average number of enzymes per bead (AEB) of samples were interpolated onto the calibrator curve constructed by AEB measurements.

#### Immunofluorence analysis in muscle

Muscles were isolated from mice that were the indicated age and genotype and immediately fixed in 4% paraformaldehyde (PFA) for 15 min at RT. Skeletal muscles were further dissected into muscle bundles of approximately 20 myofibers each. The samples were quenched in 50 mM NH_4_Cl for 30 min at RT and then saturated for 2 h in blocking solution [15% vol/vol goat serum, 2% wt/vol bovine serum albumin (BSA), 0.25% wt/vol gelatin, and 0.2% wt/vol glycine in phosphate-buffered saline (PBS) containing 0.5% Triton X-100]. Incubation with primary antibodies against Kv3.1 (1:200), Kv3.4 (1:200), and DHPR (1:200) was carried out for at least 48 h in blocking solution. The muscles were then thoroughly washed and incubated with a secondary antibody conjugated with Alexa-555 or Alexa-488 diluted in blocking solution. Images were collected with a Leica SP5 confocal microscope (Leica Microsystems, Wetzlar, Germany). The laser excitation line, power intensity, and emission range were chosen according to each fluorophore in different samples to minimize bleedthrough. Images were analyzed with the ImageJ program using the ‘plot profile’ function on a determined area of the sample. For the transverse sections, tissues were flash frozen in isopentane precooled in liquid nitrogen and embedded in an optimal cutting temperature (OCT). Cross Sect. (10 μm thick) were cut with a cryostat (CM1850 UV, Leica Microsystems, Wetzlar, Germany) and processed for the analysis of Kv3.1 (1:300), Kv3.4 (1:300). Fibre typing was analyzed in 10 μm quadriceps cryosections by immunofluorescence using combinations of the following monoclonal antibodies distributed by DSHB: BA-D5 (MyHC-I; 1:300), BF-F3(MyHC-IIb; 1:300), SC-71 (MyHC-IIa; 1:300) and not stained myofibers were MyHC-IIx. The images were captured with a Leica DFC300- FX digital charge-coupled device camera using Leica DC Viewer software, and morphometric analyses were performed using ImageJ.

#### NADH staining

For muscle histology, tissues were flash-frozen in liquid nitrogen and embedded in optimal cutting temperature (OCT) compound. Cross-Sect. (10 μm thick) were cut with a cryostat (CM1850 UV, Leica Microsystems, Wetzlar, Germany) and processed for nicotinamide adenine dinucleotide (NADH) staining.

#### Hematoxylin-eosin staining

Slides were incubated in hematoxylin for 3 min, and rinsed in water. They were then stained with eosin, dehydrated through gradient ethanol, then immersed in xylene for 10 min followed by mounting in Permount (Electron Microscopy Science, PA).

#### Pixel-to-micrometer conversion

NMJ (microscope: Leica, Thunder): 1.291 μm/pixel; GFAP & Iba1 / Nissl: (microscope: Olympus, BX51): 2.12 μm/pixel. For GFAP and Iba1 imaging conditions, all samples were stained and imaged under identical conditions on the same day / same time. We applied all black balance for background removal. Imaging conditions were as follows: GFAP (Green fluorescence):100ms; Iba1 (Red fluorescence): 50ms; Gain: 300%; Histogram: 0-200.

### Toxin, BaCl_2_ injections and nerve resection

#### Botulinum injections

0.02 units/gram BoNT was injected to the left hind limb of mice, between the Tibialis Anterior and Gastrocnemius muscles on 3months old male mice. Tissues were collected at four time points: 2-, 7-, 14-, and 30-days post injection. In addition, four mice were used as controls and received a vehicle solution injection composed of physiological saline. Tissues were collected from the control mice after 30 days.

#### BaCl_2_ injections

16 mice had an intramuscular injection of 1.2% BaCl_2_ on both Tibialis Anterior muscles. Tissues were collected at three time points: 2-, 7-, 14-, and 30-days post-injection. Four mice were included in each group, while 4 additional mice were used as controls and did not receive any injections.

#### Sciatic nerve resection

Mice were anesthetized by isoflurane inhalation, and the sciatic nerve was exposed after shaving.

the skin and making a 0.2-cm-long incision on lateral side of the left hindlimb. A 0.5 cm-long piece of sciatic nerve was removed, to prevent nerve regeneration. The incision was closed with surgical clips. Mice were sacrificed 7 days after the denervation procedure by cervical dislocation, and muscles were dissected for subsequent analyses. Skeletal muscles from the right hindlimb were sham-operated and served as a control for subsequent analyses.

#### Electrophysiological recordings

The modulatory effects of AUT00201 were studied on whole-cell currents mediated by recombinant human Kv3.1b. Manual patch clamp techniques were performed on HEK cells stably expressing Kv3.1b, In brief, cells were maintained in minimum essential medium (Sigma) with 10% fetal bovine serum (Sigma), 2 mM L-glutamine (Sigma), 10 ml/l penicillin-streptomycin (Sigma) and 0.5 mg/ml geneticin (Biowest) and maintained in a 5% CO2 incubator at 37 °C. HEK cells were grown on coverslips 18–24 h preceding recordings and transferred to extracellular solution (137 mM NaCl, 1.8 mM CaCl2, 1 mM MgCl2 4 mM KCl, 10 mM HEPES, and 10 mM glucose, pH 7.4) Recordings were made in the whole cell configuration, using an Multiclamp 700B amplifier (Axon instruments, Foster City, CA). The patch electrodes had a resistance of 2–3 MΩ when filled with intracellular solution (110 mM KCl, 0.2 mM EGTA, and 40 mM HEPES, 1 mM MgCl2, 0.1 mM CaCl2, 5 mM Na2phosphocreatine, pH 7.2). All data were low pass filtered at 10–15 kHz and digitized using Digidata 1440 A interface (Molecular Devices LLC, Sunnyvale, CA, USA).

Conductance values were obtained by dividing current by the electrochemical driving force (IK / (Vm-Ek)). Normalized conductance-voltage plots were obtained by normalizing conductance (G) to maximal conductance (Gmax) and fit using the Boltzmann isoform G = Gmax / [1 + exp ((V - V1/2) / k)], where V1/2 is the voltage at half-maximal activation and k is the slope factor.

Ionworks Quattro™ was used to assess the effects of AUT00201 on human recombinant Kv3.1a channels stably expressed in HEK cells, human recombinant Kv3.2a channels expressed in a CHO cell line and on human recombinant Kv3.4a expressed in a HEK cell. Briefly, effects of AUT00201 were tested using 384-well population patch-clamp plates. Seal resistance was measured for each well and cells were perforated by incubation with 100 µg/mL amphotericin B (Sigma-Aldrich). Cells were held at -70 mV, stepped to -15 mV for 100 ms (partial channel activation) and after 100 ms at -70 mV a second pulse to + 40 mV was applied for 50 ms (full channel activation). In all experiments, this voltage protocol was applied to cells before and following a 3 min incubation with AUT00201. AUT00201 was tested at different concentrations ranging from 50nM up to 50µM (11 points per curve). 1-cyclohexyl-1-[(7,8-dimethyl-2-oxo-1 H-quinolin-3-yl)methyl]-3- phenylurea (10µM, Aurora Screening Library), which we had previously found to be a potent and full activator of human Kv3.1 and Kv3.2 channels, was included in all assays as a standard. External buffer with the addition of dimethyl sulfoxide (0.5% DMSO, Sigma-Aldrich) was also tested to provide a vehicle baseline. Recordings were performed in the following buffers: Dulbecco’s PBS with 0.5 mM MgCl2 and 0.9 mM CaCl2 (Invitrogen) as extracellular solution and 50 mM KCl, 100 mM K-gluconate, 3.2 mM MgCl2, and 5 mM HEPES, pH 7.3 adjusted with KOH as intracellular solution (Sigma-Aldrich). An online correction of + 15–20 mV was applied to correct for junction potentials. The current signal was sampled at 10 kHz. Paired comparisons between pre- and post-drug additions of steady-state currents measured at -15 mV voltage step were used to determine the effect of the compound. Concentration-response data were normalised to the maximum effect (i.e. 100%) observed with the standard.

### Pharmacokinetics

The pharmacokinetics study was designed to assess the pharmacokinetics of AUT00201. AUT00201 was administered orally to male C57Bl/6 N (*n* = 3) as a solution in Gelucire 44/14: PEG400: Water, at a ratio of 50:30:20 [v/v/v], at the doses 3, 5 and 20 mg/kg. Pharmacokinetics experiments were carried out by Evotec (Verona, Italy) in accordance with national legislation regarding animal welfare (Italian Legislative Decree no. 26/2014 and European Directive no 2010/63/UE). All experiments were approved by the internal Aptuit Committee on Animal Research and Ethics under authorisation issued by the Italian Ministry of Health (Italian Ministry of Health Authorization Project No. 38200).

### Statistical analysis

To compare the mean difference of a dependent variable between independent groups, two-funsiisample t-tests were applied for comparisons involving two groups, while one-way analysis of variance (ANOVA) was used for comparisons involving more than two groups. ANOVAs, follow-up Tukey’s honest significant difference post hoc tests were conducted for pairwise comparisons for RT-PCR. In the preclinical study, the statistical significance of the results was analyzed using one-way ANOVA followed by LSD post-hoc analysis. All values are presented as ± standard error of the mean (SEM) and differences are statistically significant at the *p* < 0.05 level. Data normality was evaluated using the Shapiro–Wilk test.

## Electronic supplementary material

Below is the link to the electronic supplementary material.

Supplementary Material 1

## Data Availability

No datasets were generated or analysed during the current study.

## References

[1] Ajroud-Driss S, Siddique T (2015) Sporadic and hereditary amyotrophic lateral sclerosis (ALS). Biochimica et biophysica acta (BBA) - Molecular basis of disease. 1852:679–684 10.1016/j.bbadis.2014.08.010 25193032

[2] Chia R, Chiò A, Traynor BJ (2018) Novel genes associated with amyotrophic lateral sclerosis: diagnostic and clinical implications. Lancet Neurol 17:94–102 29154141 10.1016/S1474-4422(17)30401-5 PMC5901717

[3] van Es MA, Hardiman O, Chio A, Al-Chalabi A, Pasterkamp RJ, Veldink JH et al (2017) Amyotrophic lateral sclerosis. Lancet 390:2084–2098 28552366 10.1016/S0140-6736(17)31287-4

[4] Nijs M, Van Damme P (2024) The genetics of amyotrophic lateral sclerosis. Curr Opin Neurol 37:560–569 38967083 10.1097/WCO.0000000000001294 PMC11377058

[5] Rosen DR, Siddique T, Patterson D, Figlewicz DA, Sapp P, Hentati A et al (1993) Mutations in cu/zn superoxide dismutase gene are associated with Familial amyotrophic lateral sclerosis. Nature 362:59–62 8446170 10.1038/362059a0

[6] Kennedy WR, Alter M, Sung JH (1968) Progressive proximal spinal and bulbar muscular atrophy of late onset. A sex-linked recessive trait. Neurology 18:671–680 4233749 10.1212/wnl.18.7.671

[7] La Spada AR, Wilson EM, Lubahn DB, Harding AE, Fischbeck KH (1991) Androgen receptor gene mutations in X-linked spinal and bulbar muscular atrophy. Nature 352:77–79 2062380 10.1038/352077a0

[8] Katsuno M, Adachi H, Kume A, Li M, Nakagomi Y, Niwa H et al (2002) Testosterone reduction prevents phenotypic expression in a Transgenic mouse model of spinal and bulbar muscular atrophy. Neuron 35:843–854 12372280 10.1016/s0896-6273(02)00834-6

[9] Hashizume A, Fischbeck KH, Pennuto M, Fratta P, Katsuno M (2020) Disease mechanism, biomarker and therapeutics for spinal and bulbar muscular atrophy (SBMA). J Neurol Neurosurg Psychiatry 91:1085–1091 32934110 10.1136/jnnp-2020-322949

[10] Brunet A, Stuart-Lopez G, Burg T, Scekic-Zahirovic J, Rouaux C (2020) Cortical circuit dysfunction as a potential driver of amyotrophic lateral sclerosis. Front Neurosci 14:363 32410944 10.3389/fnins.2020.00363 PMC7201269

[11] Manzano R, Sorarú G, Grunseich C, Fratta P, Zuccaro E, Pennuto M et al (2018) Beyond motor neurons: expanding the clinical spectrum in kennedy’s disease. J Neurol Neurosurg Psychiatry 89:808–812 29353237 10.1136/jnnp-2017-316961 PMC6204939

[12] Shefner JM, Musaro A, Ngo ST, Lunetta C, Steyn FJ, Robitaille R et al (2023) Skeletal muscle in amyotrophic lateral sclerosis. Brain 146:4425–4436 37327376 10.1093/brain/awad202 PMC10629757

[13] Menon P, Geevasinga N, van den Bos M, Yiannikas C, Kiernan MC, Vucic S (2017) Cortical hyperexcitability and disease spread in amyotrophic lateral sclerosis. Eur J Neurol 24:816–824 28436181 10.1111/ene.13295

[14] Oki K, Halievski K, Vicente L, Xu Y, Zeolla D, Poort J et al (2015) Contractile dysfunction in muscle May underlie androgen-dependent motor dysfunction in spinal bulbar muscular atrophy. J Appl Physiol (1985) 118:941–952 25663674 10.1152/japplphysiol.00886.2014 PMC4385878

[15] Shibuya K, Misawa S, Uzawa A, Sawai S, Tsuneyama A, Suzuki Y-I et al (2020) Split hand and motor axonal hyperexcitability in spinal and bulbar muscular atrophy. J Neurol Neurosurg Psychiatry 91:1189–1194 32934003 10.1136/jnnp-2020-324026

[16] Xu Y, Halievski K, Henley C, Atchison WD, Katsuno M, Adachi H et al (2016) Defects in neuromuscular transmission May underlie motor dysfunction in spinal and bulbar muscular atrophy. J Neurosci 36:5094–5106 27147661 10.1523/JNEUROSCI.3485-15.2016 PMC4854970

[17] Abbott GW, Butler MH, Bendahhou S, Dalakas MC, Ptacek LJ, Goldstein SA (2001) MiRP2 forms potassium channels in skeletal muscle with Kv3.4 and is associated with periodic paralysis. Cell 104:217–231 11207363 10.1016/s0092-8674(01)00207-0

[18] Rudy B, Chow A, Lau D, Amarillo Y, Ozaita A, Saganich M et al (1999) Contributions of Kv3 channels to neuronal excitability. Ann N Y Acad Sci 868:304–343 10414303 10.1111/j.1749-6632.1999.tb11295.x

[19] Kaczmarek LK, Zhang Y (2017) Kv3 channels: enablers of rapid firing, neurotransmitter release, and neuronal endurance. Physiol Rev 97:1431–1468 28904001 10.1152/physrev.00002.2017 PMC6151494

[20] Rudy B, McBain CJ (2001) Kv3 channels: voltage-gated K + channels designed for high-frequency repetitive firing. Trends Neurosci 24:517–526 11506885 10.1016/s0166-2236(00)01892-0

[21] Weiser M, Vega-Saenz de Miera E, Kentros C, Moreno H, Franzen L, Hillman D et al (1994) Differential expression of Shaw-related K + channels in the rat central nervous system. J Neurosci 14:949–972 8120636 10.1523/JNEUROSCI.14-03-00949.1994 PMC6577540

[22] Vullhorst D, Klocke R, Bartsch JW, Jockusch H (1998) Expression of the potassium channel KV3.4 in mouse skeletal muscle parallels fiber type maturation and depends on excitation pattern. FEBS Lett 421:259–262 9468318 10.1016/s0014-5793(97)01577-9

[23] Ho CS, Grange RW, Joho RH (1997) Pleiotropic effects of a disrupted K + channel gene: reduced body weight, impaired motor skill and muscle contraction, but no seizures. Proc Natl Acad Sci U S A 94:1533–1538 9037088 10.1073/pnas.94.4.1533 PMC19826

[24] King EC, Patel V, Anand M, Zhao X, Crump SM, Hu Z et al (2017) Targeted deletion of Kcne3 impairs skeletal muscle function in mice. FASEB J 31:2937–2947 28356343 10.1096/fj.201600965RR PMC5472403

[25] Schiaffino S, Reggiani C (2011) Fiber types in mammalian skeletal muscles. Physiol Rev 91:1447–1531 22013216 10.1152/physrev.00031.2010

[26] Marchioretti C, Zanetti G, Pirazzini M, Gherardi G, Nogara L, Andreotti R et al (2023) Defective excitation-contraction coupling and mitochondrial respiration precede mitochondrial Ca2 + accumulation in spinobulbar muscular atrophy skeletal muscle. Nat Commun 14:602 36746942 10.1038/s41467-023-36185-w PMC9902403

[27] Rossetto O, Pirazzini M, Montecucco C (2014) Botulinum neurotoxins: genetic, structural and mechanistic insights. Nat Rev Microbiol 12:535–549 24975322 10.1038/nrmicro3295

[28] Tierney MT, Sacco A (2016) Inducing and evaluating skeletal muscle injury by notexin and barium chloride. Methods Mol Biol 1460:53–60 27492165 10.1007/978-1-4939-3810-0_5

[29] Me G, Ay HP, Mc C, Cy DC, Dd P A, Motor neuron degeneration in mice that express a human Cu,Zn superoxide dismutase mutation. Science (New York, NY) [Internet]. 1994 [cited 2024 Jun 7];264. Available from: https://pubmed.ncbi.nlm.nih.gov/8209258/10.1126/science.8209258 8209258

[30] Chivet M, Marchioretti C, Pirazzini M, Piol D, Scaramuzzino C, Polanco MJ et al (2020) Polyglutamine-Expanded androgen receptor alteration of skeletal muscle homeostasis and myonuclear aggregation are affected by sex, age and muscle metabolism. Cells 9:325 32019272 10.3390/cells9020325 PMC7072234

[31] Gegenhuber B, Tollkuhn J (2020) Signatures of sex: sex differences in gene expression in the vertebrate brain. Wiley Interdiscip Rev Dev Biol 9:e348 31106965 10.1002/wdev.348 PMC6864223

[32] Brown MR, El-Hassar L, Zhang Y, Alvaro G, Large CH, Kaczmarek LK (2016) Physiological modulators of Kv3.1 channels adjust firing patterns of auditory brain stem neurons. J Neurophysiol 116:106–121 27052580 10.1152/jn.00174.2016 PMC4961756

[33] Rosato-Siri MD, Zambello E, Mutinelli C, Garbati N, Benedetti R, Aldegheri L et al (2015) A novel modulator of Kv3 potassium channels regulates the firing of Parvalbumin-Positive cortical interneurons. J Pharmacol Exp Ther 354:251–260 26085652 10.1124/jpet.115.225748

[34] Loeffler T, Schilcher I, Flunkert S, Hutter-Paier B (2020) Neurofilament-Light chain as biomarker of neurodegenerative and rare diseases with high translational value. Front Neurosci 14:579 32595447 10.3389/fnins.2020.00579 PMC7300175

[35] Barreto-Núñez R, Béland L-C, Boutej H, Picher-Martel V, Dupré N, Barbeito L et al (2024) Chronically activated microglia in ALS gradually lose their immune functions and develop unconventional proteome. Glia 72:1319–1339 38577970 10.1002/glia.24531

[36] Levine JB, Kong J, Nadler M, Xu Z (1999) Astrocytes interact intimately with degenerating motor neurons in mouse amyotrophic lateral sclerosis (ALS). Glia 28:215–224 10559780

[37] Vargas MR, Johnson JA (2010) Astrogliosis in amyotrophic lateral sclerosis: role and therapeutic potential of astrocytes. Neurotherapeutics 7:471–481 20880509 10.1016/j.nurt.2010.05.012 PMC2967019

[38] Boillée S, Yamanaka K, Lobsiger CS, Copeland NG, Jenkins NA, Kassiotis G et al (2006) Onset and progression in inherited ALS determined by motor neurons and microglia. Science 312:1389–1392 16741123 10.1126/science.1123511

[39] Yamanaka K, Chun SJ, Boillee S, Fujimori-Tonou N, Yamashita H, Gutmann DH et al (2008) Astrocytes as determinants of disease progression in inherited amyotrophic lateral sclerosis. Nat Neurosci 11:251–253 18246065 10.1038/nn2047 PMC3137510

[40] McCrossan ZA, Lewis A, Panaghie G, Jordan PN, Christini DJ, Lerner DJ et al (2003) MinK-related peptide 2 modulates Kv2.1 and Kv3.1 potassium channels in mammalian brain. J Neurosci 23:8077–8091 12954870 10.1523/JNEUROSCI.23-22-08077.2003 PMC6740484

[41] Zhang Y, Kaczmarek LK (2016) Kv3.3 potassium channels and spinocerebellar ataxia. J Physiol 594:4677–4684 26442672 10.1113/JP271343 PMC4983625

[42] Muona M, Berkovic SF, Dibbens LM, Oliver KL, Maljevic S, Bayly MA et al (2015) A recurrent de Novo mutation in KCNC1 causes progressive myoclonus epilepsy. Nat Genet 47:39–46 25401298 10.1038/ng.3144 PMC4281260

[43] Miranda DR, Reed E, Jama A, Bottomley M, Ren H, Rich MM et al (2020) Mechanisms of altered skeletal muscle action potentials in the R6/2 mouse model of huntington’s disease. Am J Physiol Cell Physiol 319:C218–C232 32432924 10.1152/ajpcell.00153.2020 PMC7468886

[44] Saudou F, Humbert S (2016) The biology of Huntingtin. Neuron 89:910–926 26938440 10.1016/j.neuron.2016.02.003

[45] Urrutia J, Arrizabalaga-Iriondo A, Sanchez-Del-Rey A, Martinez-Ibargüen A, Gallego M, Casis O et al (2024) Therapeutic role of voltage-gated potassium channels in age-related neurodegenerative diseases. Front Cell Neurosci 18:1406709 38827782 10.3389/fncel.2024.1406709 PMC11140135

[46] Ditsworth D, Maldonado M, McAlonis-Downes M, Sun S, Seelman A, Drenner K et al (2017) Mutant TDP-43 within motor neurons drives disease onset but not progression in amyotrophic lateral sclerosis. Acta Neuropathol 133:907–922 28357566 10.1007/s00401-017-1698-6 PMC5427168

[47] Yu Z, Dadgar N, Albertelli M, Gruis K, Jordan C, Robins DM et al (2006) Androgen-dependent pathology demonstrates myopathic contribution to the Kennedy disease phenotype in a mouse knock-in model. J Clin Invest 116:2663–2672 16981011 10.1172/JCI28773 PMC1564432

[48] Katsuno M, Adachi H, Doyu M, Minamiyama M, Sang C, Kobayashi Y et al (2003) Leuprorelin rescues polyglutamine-dependent phenotypes in a Transgenic mouse model of spinal and bulbar muscular atrophy. Nat Med 9:768–773 12754502 10.1038/nm878

[49] Shahim P, Norato G, Sinaii N, Zetterberg H, Blennow K, Chan L et al (2024) Neurofilaments in sporadic and Familial amyotrophic lateral sclerosis: A systematic review and Meta-Analysis. Genes (Basel) 15:496 38674431 10.3390/genes15040496 PMC11050235

[50] Verde F, Steinacker P, Weishaupt JH, Kassubek J, Oeckl P, Halbgebauer S et al (2019) Neurofilament light chain in serum for the diagnosis of amyotrophic lateral sclerosis. J Neurol Neurosurg Psychiatry 90:157–164 30309882 10.1136/jnnp-2018-318704

[51] Poesen K, De Schaepdryver M, Stubendorff B, Gille B, Muckova P, Wendler S et al (2017) Neurofilament markers for ALS correlate with extent of upper and lower motor neuron disease. Neurology 88:2302–2309 28500227 10.1212/WNL.0000000000004029

[52] Gagliardi D, Meneri M, Saccomanno D, Bresolin N, Comi GP, Corti S (2019) Diagnostic and prognostic role of blood and cerebrospinal fluid and blood neurofilaments in amyotrophic lateral sclerosis: A review of the literature. Int J Mol Sci 20:4152 31450699 10.3390/ijms20174152 PMC6747516

[53] Gagliardi D, Faravelli I, Meneri M, Saccomanno D, Govoni A, Magri F et al (2021) Diagnostic and prognostic value of CSF neurofilaments in a cohort of patients with motor neuron disease: A cross-sectional study. J Cell Mol Med 25:3765–3771 33609080 10.1111/jcmm.16240 PMC8051694

[54] Zucchi E, Bedin R, Fasano A, Fini N, Gessani A, Vinceti M et al (2018) Cerebrospinal fluid neurofilaments May discriminate upper motor neuron syndromes: A pilot study. Neurodegener Dis 18:255–261 30428468 10.1159/000493986

[55] Benatar M, Ostrow LW, Lewcock JW, Bennett F, Shefner J, Bowser R et al (2024) Biomarker qualification for neurofilament light chain in amyotrophic lateral sclerosis: theory and practice. Ann Neurol 95:211–216 38110839 10.1002/ana.26860 PMC10842825

[56] Clarke BE, Patani R (2020) The microglial component of amyotrophic lateral sclerosis. Brain 143:3526–3539 33427296 10.1093/brain/awaa309 PMC7805793

[57] Sadeghdoust M, Das A, Kaushik DK (2024) Fueling neurodegeneration: metabolic insights into microglia functions. J Neuroinflammation 21:300 39551788 10.1186/s12974-024-03296-0 PMC11571669

[58] Pehar M, Harlan BA, Killoy KM, Vargas MR (2017) Role and therapeutic potential of astrocytes in amyotrophic lateral sclerosis. Curr Pharm Des 23:5010–5021 28641533 10.2174/1381612823666170622095802 PMC5740017

[59] Neusch C, Bähr M, Schneider-Gold C (2007) Glia cells in amyotrophic lateral sclerosis: new clues to Understanding an old disease? Muscle Nerve 35:712–724 17373702 10.1002/mus.20768

