# Supplementary material for: Kv3 channel agonist ameliorates the phenotype of a mouse model of amyotrophic lateral sclerosis
Source: Acta Neuropathol Commun. 2025 Jul 14;13:153. doi: 10.1186/s40478-025-02067-z (PMC12257725; doi:10.1186/s40478-025-02067-z)
Supplement: Supplementary file 1 — Supplementary Material 1 [file 40478_2025_2067_MOESM1_ESM.docx]

**Kv3 channel agonist ameliorates the phenotype of a mouse model of amyotrophic lateral sclerosis**

Manuela Marabita^1,2^*, Caterina Marchioretti^3,4^*, Aishwarya Aravamudhan^3,4^*, Simona Zito^3,4^, Antonella Falconieri^3,4^, Emanuela Zuccaro^3,4^, Roberta Andreotti^3,4^, Lisa Gambarotto^5^, Samuele Metti^5^, Marika Tonellato^3^, Valentina Adami^1,2^, Kyung Ho Park^6^, Martin J. Gunthorpe^7^, Charles H. Large^7^, Agostino Marasco^1,2^, Sara Vianello^8^, Jessica Rosati^9,10^, Elisa Belluzzi^11,12^, Assunta Pozzuoli^11,12^, Carlo Biz^11^, Pietro Ruggieri^11^, Manuela Basso^13^, Angelo Poletti^14^, Giuseppe Alvaro^1,2^, Gianni Sorarù^8^, Paolo Bonaldo^5^, Ornella Rossetto^3^, Nadia Pilati^1,2^#, Maria Pennuto^3,4^#

^1^ Autifony Srl, Istituto di Ricerca Pediatrica Città della Speranza, 35127, Padova, Italy;

^2^ BioTiChe, 35127, Padova, Italy;

^2^ Department of Biomedical Sciences (DBS), University of Padova, 35131, Padova, Italy;

^3^ Veneto Institute of Molecular Medicine (VIMM), 35100, Padova, Italy;

^4^ Department of Molecular Medicine (DMM), University of Padova, 35131, Padova, Italy;

^5^ Naason Science Inc, Saengmyung-Ro 123, Osong-eup, Heungdeok-gu, Cheongju-si, Chungbuk, Korea 28160;

^6^ Autifony Therapeutics, Ltd, Stevenage Bioscience Catalyst, Gunnels Wood Road, Stevenage, SG1 2FX, UK;

^7^ Department of Neuroscience (DNS), Neuromuscular Center, University of Padova, 35128 Padova, Italy;

^8^ Cellular Reprogramming Unit, Fondazione IRCCS Casa Sollievo della Sofferenza, 71100 Foggia, Italy;

^9^ UniCamillus - Saint Camillus International University of Health Sciences, Via di Sant’Alessandro, 8- 00131 Rome, Italy.

^10^ Department of Orthopaedics and Orthopaedic Oncology, Department of Surgery, Oncology, and Gastroenterology (DiSCOG), University-Hospital of Padova, 35128 Padova, Italy;

^11^ Musculoskeletal Pathology and Oncology Laboratory, Department of Surgery, Oncology, and Gastroenterology (DiSCOG), University of Padova, 35128 Padova, Italy;

^12^ Department of Cellular, Computational and Integrative Biology (CIBIO), University of Trento, 38123, Trento, Italy;

^13^ Department of Biomolecular and Pharmacological Sciences Rodolfo Paoletti, University of Milan, 20133, Milan, Italy.

* Equal contribution

# Corresponding authors

Maria Pennuto, PhD

[maria.pennuto@unipd.it](mailto:maria.pennuto@unipd.it)

Nadia Pilati, PhD

[nadia.pilati@biotiche.com](mailto:nadia.pilati@biotiche.com)

**Supplementary** **Material**

**Supplementary Tables**

**Supplementary Table 1**. Description of control subjects and ALS patients.

**Supplementary Table 2.** Primers used for quantitative real-time PCR analyses.

**Supplementary Figures**

**Supplementary Fig. 1.** Analysis of the effect of acute motor unit damage on gene expression.

**Supplementary Fig. 2.** Treatment with an agonist of Kv3 channels does not modify the phenotype of AR100Q mice.

**Supplementary Fig. 3.** Treatment of SOD1-G93A mice with AUT00201 does not modify NMJ pathology.

**Supplementary Fig. 4**. Analysis of Kcnc4 expression in the skeletal muscle of patients suffering from ALS.

**Supplementary Tables**

**Supplementary Table 1**. Description of control subjects and ALS patients.

| **Control subjets** | **Disease** | **Muscle** |
| --- | --- | --- |
| 1 | N.A. | Vastus lateralis |
| 2 | N.A. | Vastus lateralis |
| 3 | N.A. | Vastus lateralis |
| 4 | N.A. | Vastus lateralis |
| **Sporadic mutation** |  | **Muscle** |
| 1 | ALS | Vastus lateralis |
| 2 | ALS | Vastus lateralis |
| 3 | ALS | Vastus lateralis |
| 4 | ALS | Vastus lateralis |
| 5 | ALS | Vastus lateralis |
| 6 | ALS | Vastus lateralis |
| 7 | ALS | Vastus lateralis |
| 8 | ALS | Vastus lateralis |
| 9 | ALS | Vastus lateralis |
| 10 | ALS | Vastus lateralis |
| 11 | ALS | Vastus lateralis |
| 12 | ALS | Vastus lateralis |
| **TDP-45 mutation** |  |  |
| 1 | ALS | Vastus lateralis |
| **FUS mutation** |  |  |
| 1 | ALS | Vastus lateralis |
| 2 | ALS | Vastus lateralis |
| 3 | ALS | Vastus lateralis |
| **C1ORF72 mutation** |  |  |
| 1 | ALS | Vastus lateralis |
| 2 | ALS | Vastus lateralis |
| 3 | ALS | Vastus lateralis |
| 4 | ALS | Vastus lateralis |
| **SOD1 mutation** |  |  |
| 1 | ALS | Vastus lateralis |
| 2 | ALS | Vastus lateralis |
| 3 | ALS | Vastus lateralis |

**Supplementary Table 2. Primers used for quantitative real-time PCR analyses.**

| **Mouse** | **Fw** | **Rv** |
| --- | --- | --- |
| *Kcnc1* | CCCTACTCATCCCGCTACG | CTCCAGACAGAAGGTTGTGATG |
| *Kcnc2* | TCGCATGTGGGCTCTTT | AAAGGTTGTGATGGAAACCAAA |
| *Kcnc4* | TGGGCTGTGGTCACCATGAC | CTCTCGACCACACCCTCTTCC |
| *b-actin* | TGA ACC CTA AGG CCA ACC GTG AAA | GAG TCC ATC ACA ATG CCT GTG GTA |
| *Myh8* | GAGGGCATCCGCATCTG | GATGAACTGTCCCTCTGGAATAG |
| *Runx1* | CACCTACCATAGAGCCATCAAA | TACTGGTAGGACTGGTCATAGG |
| *Myh3* | GGGACCTTGCCAAGAAGAA | GTCGTTCCTCACGGTCTTG |
| *MyoG* | AGTGAATGCAACTCCCACAG | GACGTAAGGGAGTGCAGATTG |
| *Musk* | ATCACCACGCCTCTTGAAAC | TGTCTTCCACGCTCAGAATG |
| *Chrn* | AGCAGAACTGCAGCATGAA | CGCTCTCCATGAAGTTACT |
| *Atrogin* | CTTGAGGGGAAAGTGAGACG | GCAAACACTGCCACATTCT |
| **Human** | **Fw** | **Rv** |
| *KCNC4* | TTCAAGCTCACACGCCACTTCG | TGCCAAATCCCAAGGTCTGAGG |
| *ACTB* | GGACTTCGAGCAAGAGATGG | AGCACTGTGTTGGCGTACAG |


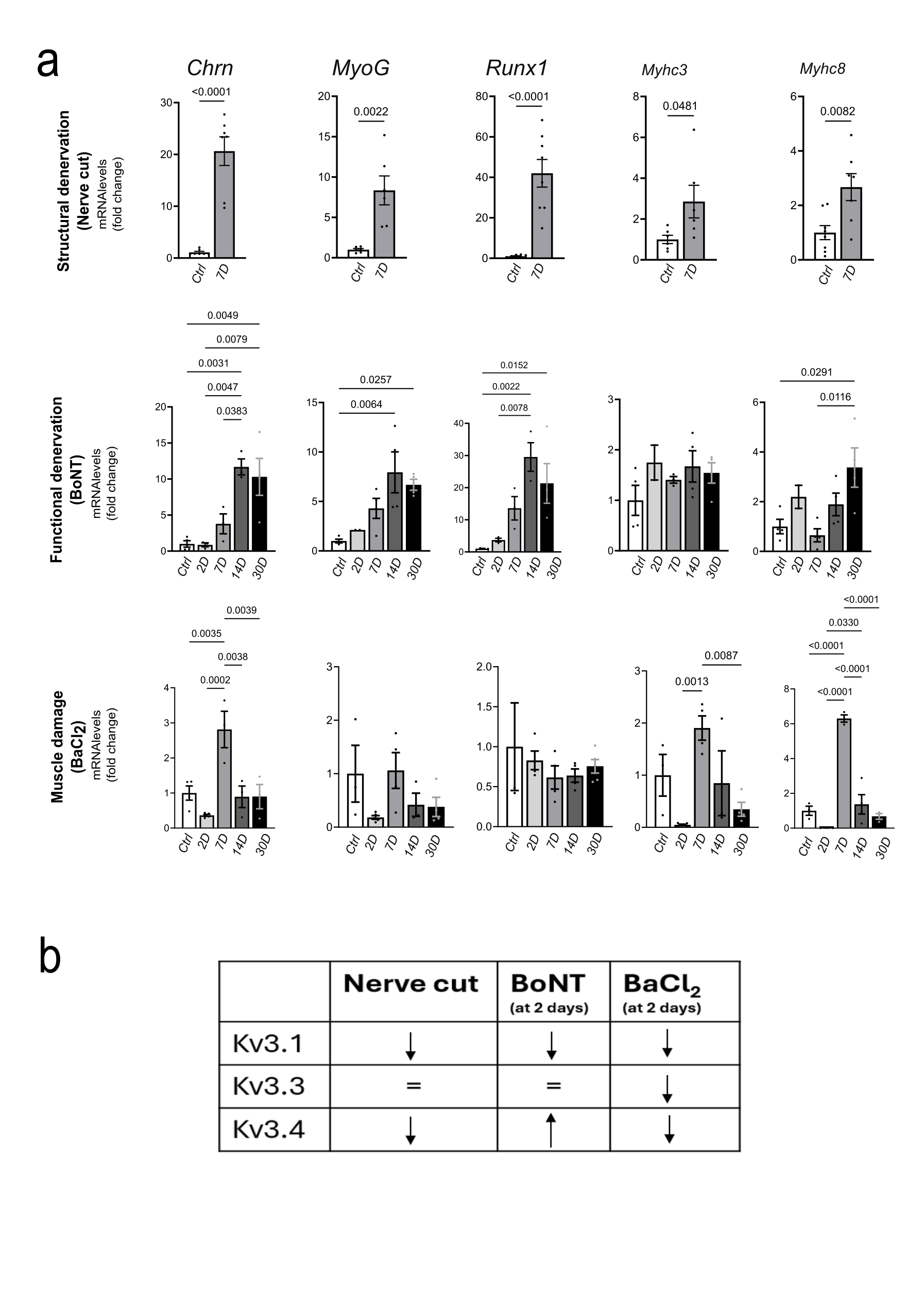


**Supplementary Fig. 1. Analysis of the effect of acute motor unit damage on gene expression.**

a. Real-time PCR analysis of the transcript levels of the indicated denervation and muscle regeneration markers normalized to beta-actin in the TA at the indicated time points.

b. Summary table showing Kv3 channels expression pattern in acute damage of the motor unit. The graphs show the mean ± SEM; significance was tested with two-way ANOVA followed by Tukey’s HSD test or by the two-tailed Student’s t-test.


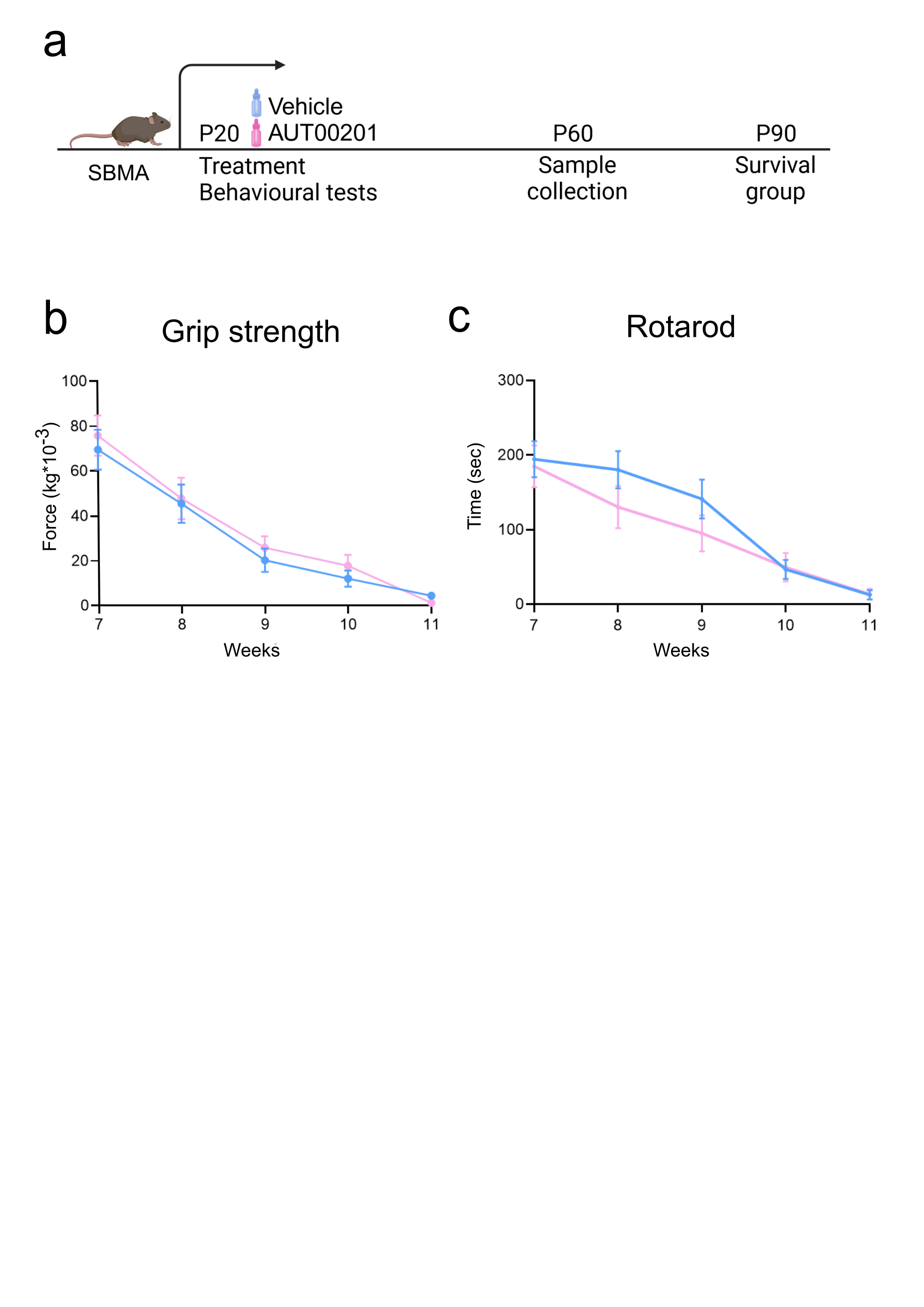


**Supplementary Figure 2. Treatment with an agonist of Kv3 channels does not modify the phenotype of AR100Q mice.**

1. Scheme of treatment and behavioral tests. Grip strength analysis of muscle force of AR100Q mice treated with either vehicle or AUT00201 (n = 8 mice/group).
2. Rotarod task analysis of motor coordination of AR100Q mice treated with either vehicle or AUT00201 (n = 8 mice/group).

The graphs show the mean ± SEM; significance was tested using one-way ANOVA followed by LSD post-hoc analysis.


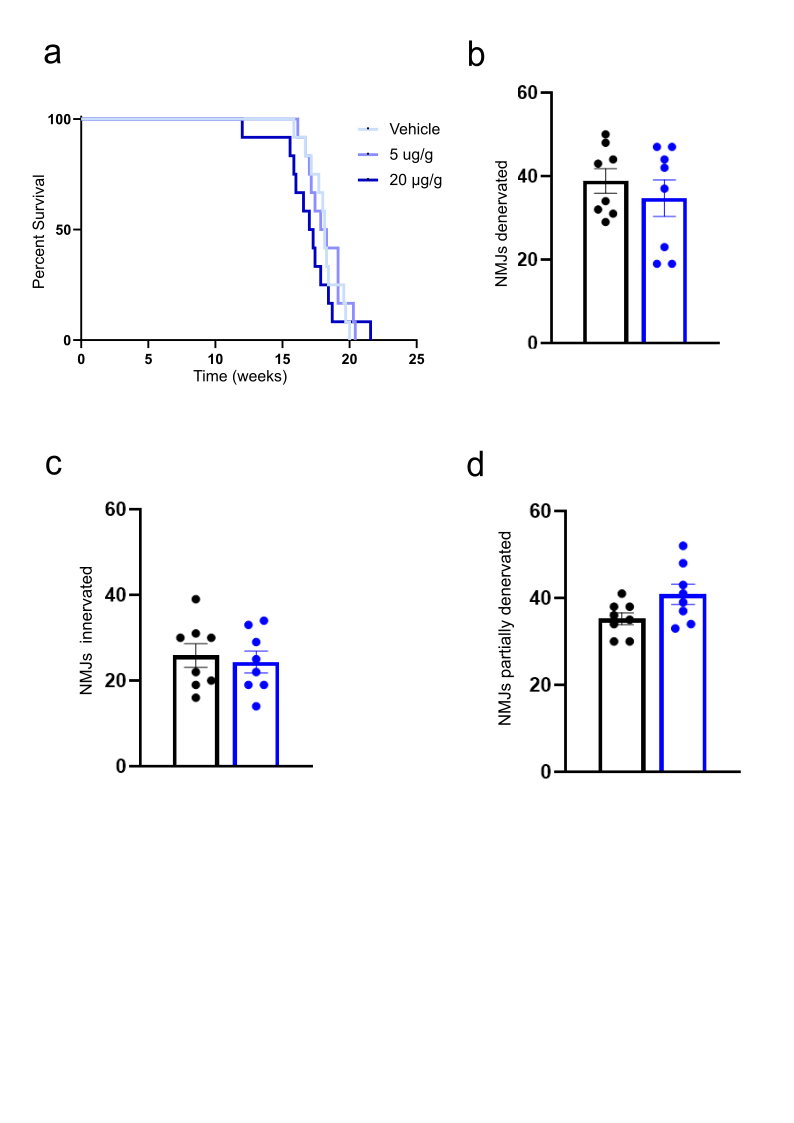


**Supplementary Figure 3. Treatment of SOD1-G93A mice with 20 µM of AUT00201 does not modify survival and NMJ pathology.**

a) Kaplan-Meier survival curves of SOD1-G93A mice (n = 12 mice/group). Survival curves were compared using Log-rank (Mantel-Cox) test.

b-c) NMJ analysis in the gastrocnemius muscle of wild type and SOD1-G93A mice. The degree of overlap between the SV2 (pre-synaptic) and α-bungarotoxin (post-synaptic) regions was divided into three different categories: ‘Fully innervated’ for completely overlapping, ‘partially innervated’ for partial overlapping, and ‘denervated’ for non-overlapping pre-synaptic and post-synaptic regions.

The graphs show the mean ± SEM; significance was tested by the two-tailed Student’s t-test.


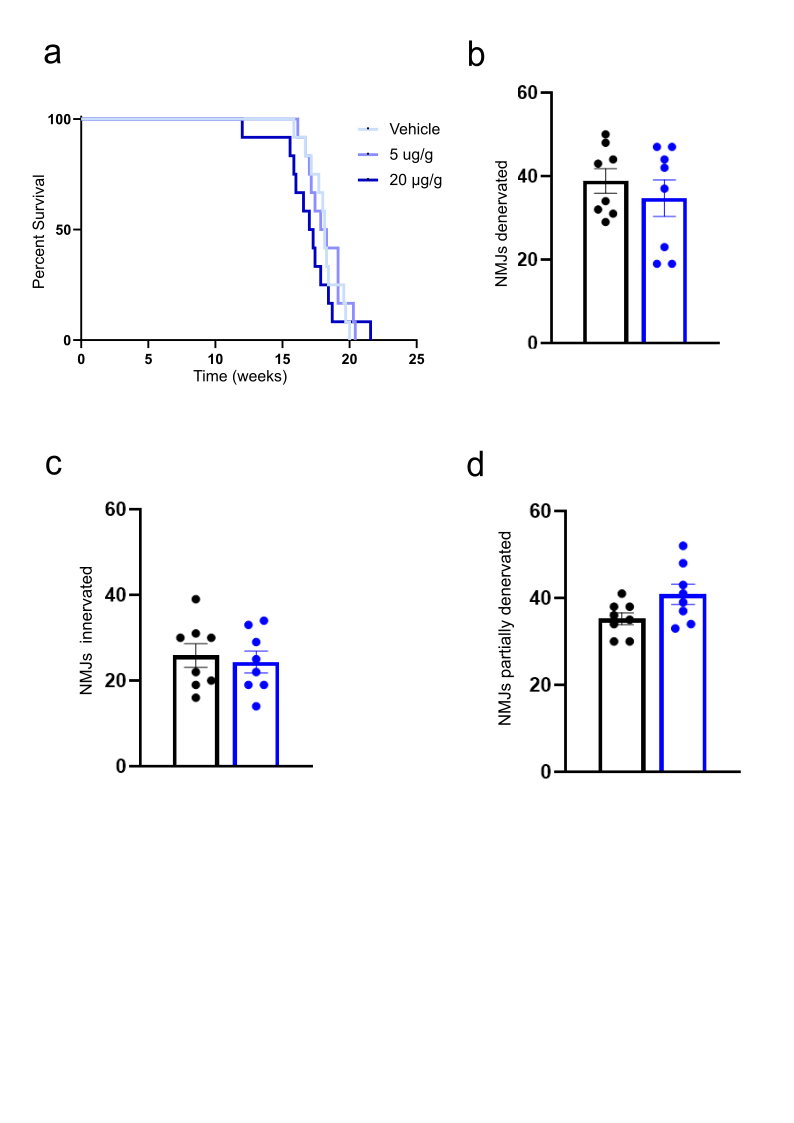


**Supplementary Figure 4. Analysis of Kcnc4 expression in the skeletal muscle of patients suffering from ALS.**

Real-time PCR analysis of the transcript levels of *Kcnc4* normalized to *beta-actin* in human muscle biopsies from different ALS patients and healthy controls (n = 1-4). The graphs show the mean ± SEM; red dots represent female subjects, and blue dots represent male subjects.
